# Pseudopotential and structural effects on electronic properties in transition metal oxides using Wannier functions

**DOI:** 10.1039/d5ra09637e

**Published:** 2026-06-05

**Authors:** Aykut Öztürk, Peter Kraus

**Affiliations:** a Technische Universität Berlin, Conductivity and Catalysis Lab Hardenbergstr. 40 10623 Berlin Germany oeztuerk@tu-berlin.de peter.kraus@tu-berlin.de

## Abstract

How do pseudopotential choices affect the electronic transport properties in first-principles calculations? How does the atomic structure affect the computed properties? In this work, we present a comparison of six different pseudopotential libraries and their impact on computed material properties, including band gap, high-frequency permittivity, and electrical conductivity, using a set of transition metal oxides. We also highlight that Wannier functions can be used to calculate electronic transport properties of materials. Our workflow demonstrates that Wannier functions provide a reliable, accurate method to handle complex systems using any pseudopotential library. We validate and compare our results with existing theoretical works and experimental values from the literature. We find that the effect of various pseudopotentials on the calculated band gap, electrical conductivity, and high-frequency permittivity is negligible. Therefore, Wannier functions can be used to benchmark calculations of band gaps and electrical conductivities of metallic and semiconducting, as well as magnetic TMOs. We also find that the effect of the choice of the atomic structure (*i.e.*, experimental *vs.* relaxed/optimized) on the electrical conductivity is not negligible. The experimental structure generally gives a more accurate result in comparison with experimental values of intrinsic conductivity than the relaxed atomic structure.

## Abstract 2

The influence of pseudopotential and structure choices on transport properties of transition metal oxides such as band gap, high frequency-permittivity, and electrical conductivity is investigated using Wannier functions.

## Graphical abstract

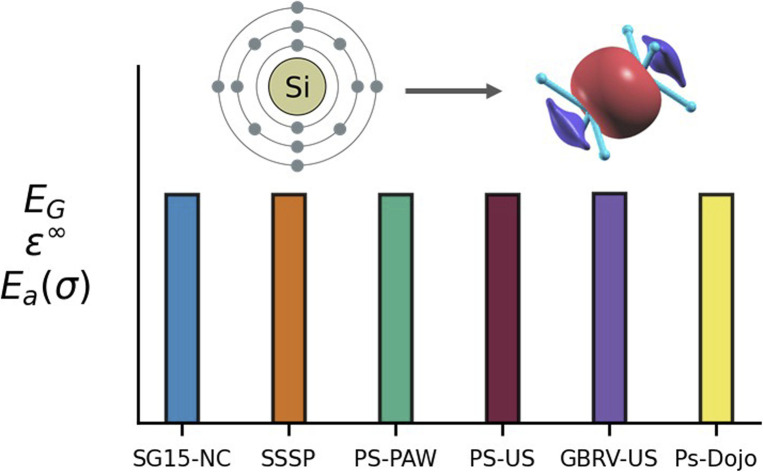

## 1 Introduction

Transition metal oxides (TMOs) have unique electronic properties and complex structures. In energy storage, TMOs serve as electrode materials, particularly as cathodes in lithium-ion batteries, due to their tunable conductivity.^1^ In electronics, TMOs are active layer materials in sensors, transistors, and memory devices, due to the tunability of their properties and optical transparency.^2^ In electrocatalysis^3^ and photocatalysis,^4^ TMOs are used as electrolytes and photocatalysts due to the strong covalent interaction between the transition metal ‘d’ orbitals and oxygen ‘p’ orbitals. This orbital interaction determines key catalytic properties, such as the reactivity of oxide surfaces and the adsorption of reactants, by facilitating charge transfer. While there are many practical applications of TMOs, *ab initio* modeling of electronic transport properties in TMOs remains challenging, due to a limited understanding of electronic behavior within these complex structures.^5^

Several recent theoretical studies have focused on calculating the electrical properties of TMOs. Borlido *et al.*^6^ reported on the effect of pseudopotentials (PPs) compared to the all-electron approach on band gap properties of solids. The all-electron approach was shown to agree very well with existing experimental band gap results. The absolute error in band gaps with respect to the experimental values can in some cases increase dramatically when PPs are used with a different exchange-correlation (XC) functional than that used for their construction. Kraus *et al.*^7^ showed that inconsistent PP – XC-functional combinations do not affect the prediction of the lattice constants in first-principles calculations. This tells us that the combination of PP and XC-functional might be critical, and this possibility will be assessed in the current work.

Petousis *et al.*^8^ reported that density functional perturbation theory (DFPT^9^) can be used to find the high-frequency permittivity of a wide range of inorganic materials (88 structures) with a band gap greater than 0.1 eV. They presented a workflow to calculate the electronic and ionic contributions to the permittivity of the materials. They stated that GGA is, in general, more accurate than LDA for finding dielectric permittivity.

Ricci *et al.*^10^ reported that electrical conductivity can be calculated from the band structure of the material using smoothed Fourier interpolation of the bands (as implemented in the BoltzTrap^11^ code) for a wide range of inorganic materials. However, the direct Fourier interpolation method is computationally costly, as a dense *k*-point mesh has to be used to obtain convergence. Instead of the direct band interpolation method, an alternative method is the Wannier-based approach, which uses maximally localized Wannier functions (MLWFs)^12^ to interpolate band structures (as implemented in the BoltzWann^13^ code). Since the BoltzTrap code uses direct Fourier interpolation of the bands to calculate electrical conductivity, it requires a denser *k*-point grid in comparison to the BoltzWann code. A comparison of the computational cost of BoltzTrap and BoltzWann, including different *k*-mesh density, is included in the SI, Section 2.3.4.

Neither of these high-throughput (HT) works^8,10^ evaluated the impact of different pseudopotential libraries on the calculated electrical properties. Both of the works used relaxed structures instead of the available experimental data. Since one of the purposes of this work is to compare theoretical data with existing experimental values from literature, we will compare the effect of the choice of atomic structure on the materials' properties, such as electrical conductivity.

Finally, Youssef *et al.*^14^ also showed that Wannier functions can accurately capture field-dependent polarization in binary oxides. They used the Berry phase approach with MLWFs to show the effect of a high electric field on defect formation energy.

In the current work, we focus on three topics: (i) the use of Wannierization as a method for band structure interpolation as applied to the calculation of electronic properties of TMOs, (ii) the effect of atomic structure (experimental or relaxed) on the electronic properties, and (iii) an assessment of the impact of PP selection by comparing results obtained with the following six PP libraries commonly used in the literature:

• Optimized Norm Conserving Vanderbilt PPs (SG15-NC^15^),

• Ultra-Soft PPs from Rutgers (GBRV-US^16^),

• Projected Augmented Wave PPs of Dal Corso (PS-PAW^17^),

• Ultra-Soft PPs of Dal Corso (PS-US^17^),

• Standard Solid-State Pseudopotential (SSSP^18^).

• PseudoDojo PPs (Ps-Dojo) from van Setten^19^

In particular, we focus on the calculation of the electronic transport properties, including the electronic band gap, high-frequency dielectric permittivity, and electrical conductivity. Our approach systematically uses MLWFs for interpolation of electronic bands, even for complex systems such as TMOs.

The TMOs systems were selected because of their importance in applications such as energy storage, electronics, and catalysis, as well as to evaluate the methodology for different polymorphs of the same formula (TiO_2_-R and TiO_2_-A), various oxidation states (MoO_2_ and MoO_3_), effects of layered structures and tunable band gap (V_2_O_5_ and V_2_O_3_), effect of magnetism in Cr_2_O_3_, and ZnO, with an experimental band gap of ∼3.4 eV which is notoriously difficult to capture by traditional density functional theory (DFT). We validate our methodology using data from existing computational datasets, including data from the Materials Project (MP^20^), and also using available experimental data. Finally, we present a study of the impact of PP selection on the electronic transport properties of selected TMOs, using the local density (LDA) as well as a generalised gradient (GGA) density functional approximation (DFAs). Additionally, we investigated the Hubbard + *U* correction with the PBE functional on electronic properties of the studied materials.

We find that our approach using MLWFs enables the calculation of high-frequency permittivity in TMOs, regardless of the choice of PPs. Another finding of this work is that the electrical conductivity of materials calculated using the BoltzWann code implemented with MLWFs is in good agreement with the results obtained from the BoltzTrap code implemented with direct interpolation by Ricci *et al.*^10^ Finally, we showed that the choice of PPs has a negligible impact on the calculated band gap, electrical conductivity, and high-frequency permittivity, while the atomic structure plays an important role in predicted electrical conductivity.

## 2 Methods

All DFT calculations were performed using Quantum ESPRESSO version 7.2 (QE)^21^ and Wannier90 version 3.1.0 (W90).^22^ All calculations were carried out using the Perdew–Zunger (PZ) LDA^23^ or the Perdew–Burke–Ernzerhof (PBE) GGA,^24^ and Hubbard corrections with +*U* values from AFLOW.^25^ Spin-unpolarized calculations were used, except for the magnetic Cr_2_O_3_, where spin-polarization was allowed. BoltzWann version 3.1.0 (post-W90)^13^ was used to calculate the electrical conductivity of materials. The PBE + *U*_AFLOW_ calculations were not possible with the SG15-NC PP library, since the SG15-NC library does not have the required orbital information for the application of Hubbard corrections. However, the PS-Dojo library is included, representing norm-conserving PPs.

### 2.1 Electronic band localization

PZ and PBE, being LDA and GGA functionals, have some known limitations, including incorrect description of non-covalent interactions as well as poor electron localization due to the self-interaction error. Therefore, it is challenging to capture localized electron–electron interactions using PZ or PBE in complex systems such as TMOs, which may exhibit strong electronic correlation.^5^ One approach to overcome this limitation is by transforming the delocalized electronic states into a localized orbital representation and then computing the electronic properties of TMOs using such localized descriptions. The result of the localization procedure strongly depends on the quality of the input wavefunction, the choice of the bands that should be localized, as well as the method by which they are localized. One option is to minimize the spread of these orbitals, *i.e.*, computing maximally localized Wannier functions (MLWFs).

A common way of constructing MLWFs is by projecting the input wavefunction onto a set of atomic or hydrogenic orbitals (*e.g.*, s, p, d, f) or their common hybridization (*e.g.*, sp, sp^2^, sp^3^d, *etc.*). These then form an initial guess for the localization procedure. If suitable projections are chosen, the localization converges quickly. However, this approach requires manual specification of the projections. Generally, some “chemical intuition” is required to ensure the initial guess is appropriate. There are two common ways of automating the construction of MLWFs: the selected columns of the density matrix (SCDM)^26^ method, and the projectability-disentangled Wannier functions (PDWFs)^27^ method. In SCDM, an optimal set of basis vectors is generated directly from the density matrix. SCDM uses all selected bands (*i.e.*, both valence and conduction bands) to construct MLWFs, so that the free electron contribution in the system can be screened. This is useful, especially for materials that have no band gap, *i.e.*, materials with a metallic character. In PDWFs, for each Bloch state (*i.e.*, the delocalized electronic wavefunctions from QE), PDWFs resemble a predefined set of physically intuitive local atomic orbitals, which are taken from the PPs used in the calculation. This is useful, especially for materials with a band gap. If the calculation is concentrated around the Fermi level, where the electronic transport happens, and if the initial guess for localized orbitals is chosen correctly, then the spread of the MLWFs will be smaller, and the calculation will be less costly. As long as the spread is small, the calculation gives a more accurate result, because Wannierization localizes well.

For the metallic systems, we employed the SCDM method in conjunction with automatic projection for the initial guesses for Wannier functions. This method enables the initialization of Wannierization without specifying frozen or disentanglement windows. We set sufficient iterations to have a localized spread of Wannier functions. The number of Wannier functions is selected based on the number of bands, keeping the metallic character of the system while generating localized orbitals to calculate the electronic conductivity of the system.

For insulators as well as magnetic cases (*E*_G_ > 1 eV), the PDWFs method was carried out manually for the selected materials, according to the workflow of Qiao *et al.*^27^ Automated projection (*e.g.* using an AiiDA workflow) will be carried out in future work focusing on benchmarking of DFAs. In this method, several terms have to be defined, including disentanglement windows, the number of Wannierized bands, and orbital projections, to construct the initial projectors. The inner (frozen) window is set to 2 eV above the conduction band minimum to reproduce states around the Fermi energy. The outer (disentanglement) window is set to the maximum and minimum eigenvalues of the system. The number of Wannierized bands is the number of projectable atomic orbitals in the selection of the disentanglement window. The orbital projections can be selected by checking the density of states representation of the bands.

### 2.2 Choice of pseudopotentials

Pseudopotentials (PPs) are used to model the atomic nuclei and core electrons of the studied system. The use of PPs is a simplification that reduces computational demands while maintaining accuracy in predicting electronic structure.^28^ PPs replace core states with an effective potential, so core states act like a point charge. This simplification makes the wavefunctions represented by a small number of plane waves, enabling efficient electronic-structure calculations.

Six different PP libraries were used in this work to investigate their effect on the electrical properties of the studied materials:

• The norm-conserving pseudopotentials (NC PPs, represented by SG15-NC) are constructed to explicitly treat valence states. The inside of the core states is replaced by using pseudo wavefunctions, while keeping the total charge (the norm) consistent with the all-electron wavefunctions. The outside of the core states in NC PPs is the same as in all-electron bases. This means the NC PPs can reproduce all-electron-like wavefunctions around the nucleus, achieving transferability, *i.e.*, the potential is similarly suitable for different chemical environments. However, NC PPs need a higher number of plane waves and a higher cut-off energy; therefore, the computational time is not as fast as other PP libraries.

• The ultra-soft pseudopotentials (US PPs, represented by GBRV-US and PS-US) relax (or “soften”) the norm-conservation constraint of the NC PPs. As a consequence, they require a lower cut-off energy. They are designed to be faster but maintain accuracy by including augmented charges localized in the core region.

• The projector-augmented wave pseudopotentials (PAW PPs, represented by PS-PAW) are based on the all-electron PAW method, which explicitly represents the interactions between the nucleus, core, and valence electrons. The US PPs and PAW PPs can both provide better transferability at lower plane-wave cut-offs than NC PPs. PAW is a mixed approach, combining ideas from US PPs^29^ and the linearized augmented-plane-wave method (LAPW^30^). Notably, in calculations within the Materials Project (MP), PAW-based PPs are used as the standard PP library.^31^ There is no conceptual difference between PS-PAW, which is used in the current work, and the PAW PPs used in the MP calculations (carried out using VASP). The only practical difference is in the valence states: in the MP calculations used as a reference in this work, the _pv variants are used (for Ti, 10 explicit electrons in the 3p, 3d, and 4s shells), while the PS-PAW PPs are more analogous to the _sv variants available in VASP (for Ti, 12 explicit electrons, including also the semi-core 3s shell), see ref. 32.

• The SSSP library is a set of PPs optimized specifically for solid-state systems (*i.e.*, bulk structures). This library enables the use of a high-performance PP for each element, based on a rigorous comparison of PPs of various construction. It includes PPs from the libraries mentioned earlier, and can be used for applications in high-throughput calculations, such as benchmarking. There are two variants of the library: precision and efficiency. The difference between these libraries comes from the benchmarking procedures, such as the equation of states (Δ-factor) or band structure deviations. The efficiency variant is more suitable for benchmarking, as it is less computationally costly than the precision variant, due to lower cut-off energies for most of the elements. In this work, we have used the SSSP-efficiency library version 1.3.0. For the materials studied, the difference between SSSP-efficiency and SSSP-precision is only in the value of the wavefunction and density cut-off.

• The PseudoDojo library (v0.5) is a set of NC PPs designed to provide high accuracy across a wide range of materials, including elemental solids and their oxides. Unlike SSSP, which is a composite library, PseudoDojo provides a consistent family of NC PPs generated using a unified methodology. The library is built using tight plane-wave cut-off energy tests, while reproducing formation energies across different chemical compounds.^19^ The PseudoDojo PP library was constructed by testing with both the PBE and the PZ DFAs.

### 2.3 Materials properties

#### 2.3.1 Band gaps

The band gap (*E*_G_) is the energy difference between the valence band maximum and the conduction band minimum. As we use MLWFs to calculate the electronic properties of TMOs, they must be able to reproduce the structures of both valence and conduction bands. Therefore, for materials with a lower band gap (<1 eV), the SCDM method was used to capture the delocalized electronic behavior in metallic systems. For materials with a higher band gap, we use fixed occupations to obtain the wavefunction, and the projection is performed using all atomic orbitals listed in the respective PP files.

The cut-off energies are taken directly from recommended values in the PP files. The highest cut-off energy among the elements in the TMO is used. For example, in the SSSP-efficiency library, the kinetic energy cut-off for oxygen wavefunctions is 50 Ry, and the kinetic energy cut-off for charge density is 400 Ry. The rest of the cut-off energies for each material can be found in the SI, Table S1. The *k*-point mesh for the system is determined by performing a convergence test for Brillouin zone sampling. The convergence criterion is based on the change of the total energy of the system with mesh density. When the total energy per atom is converged within 1 meV per atom between meshes, the smaller *k*-point mesh is selected. This ensures that the *k*-point selection will give reliable results while maintaining the speed of the calculation.

#### 2.3.2 High-frequency permittivities

The dielectric permittivity is a frequency-dependent quantity that consists of electronic and ionic contributions. However, in this work, we focus only on the electronic contribution, *i.e.*, the high-frequency part (*ε*^*∞*^). The high-frequency permittivity can be calculated from the macroscopic polarization **P** of the material as a response to an external field (*ε*) or as the second derivative of the total energy (*E*) with respect to 
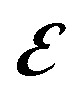
:^33^1
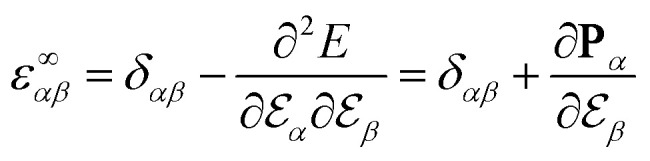
where *α* and *β* represent the indices of the coordinates of the system, and *δ*_*αβ*_ is the Kronecker delta, which equals 1 when the indices are identical (*α* = *β*), otherwise 0.

The high-frequency dielectric permittivity can be computed by applying a homogeneous electric field within DFT calculations with periodic boundary conditions. The dipole moment can also be calculated using the modern theory of polarization,^34^ where polarization is described as a Berry phase of the occupied electronic wavefunctions, see *e.g.* Spaldin.^35^ One can derive eqn (1) using the change in the dipole moment Δ**p** as a function of the change in the electric field strength: 
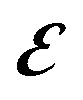
 (ref. 36)2
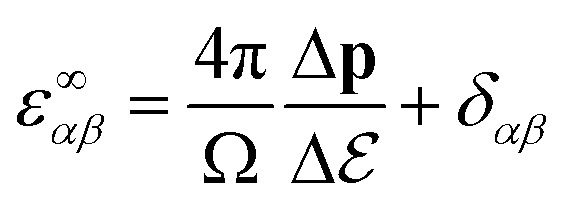
where *Ω* is the unit cell volume. Additionally, at the high frequency limit, the change in the total dipole moment Δ**p** can be replaced by the electronic component, Δ**p**_el_.

An alternative approach based on MLWFs has been proposed by Vanderbilt and Resta.^34^ Each MLWF represents a localized electron pair. Since Wannier functions are real-space representations of the constructed wavefunctions, this approach should be equivalent to the Berry phase formalism.^34^ The sum over the Wannier centers of the occupied bands gives the electronic contribution to dipole moments,3
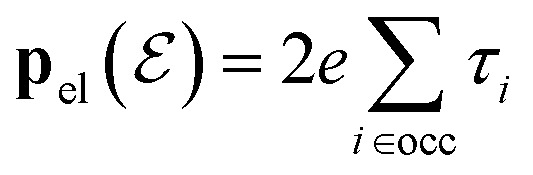
where *τ*_*i*_ represents the position vector of the *i*th Wannier center, and *e* is the elementary charge. After finding Δ**p**_el_ at two different field strengths *ε*, the high-frequency permittivity can be calculated using eqn (2).

#### 2.3.3 Electrical conductivities

In solids, electrical conductivity arises from the transport of charge carriers such as electrons, holes, or ions. In TMOs, three common conduction mechanisms describing charge transport can be expected: intrinsic electronic conduction, a small or large polaron conduction mechanism, and variable-range hopping (VRH). Intrinsic conduction is governed by delocalized carriers moving in bands from the valence band to the conduction band at high temperatures.^37^ VRH occurs by carrier transport between localized states in a disordered energy landscape at low temperatures, as described by Mott.^38^ The polaron model occurs at mid-range temperatures through electron-phonon interaction *via* hopping. A polaron can be defined as a quasiparticle consisting of an electron that is polarized around the displaced ions, leading to self-trapping. The polaron model can be broadly categorized into two different mechanisms, namely small and large polarons. A small polaron forms when the short-range electron–lattice interaction is dominant, resulting in the self-trapped state localizing to a single site. An example of small polaron conduction is TiO_2_-R.^39^ In contrast, a large polaron forms when long-range electron–lattice interaction is dominant, resulting in the self-trapped state localizing to more than a single site.^40^ In TiO_2_-A, the conduction mechanism at low temperature is driven by large-polaron conduction.^41^ However, in this work, using the MLWFs approach within the constant relaxation time approximation, we focus only on the intrinsic electronic transport regime.

The Boltzmann transport equation can be used to calculate transport properties, describing how a particle moves in a solid.^42^ Using first-principles methods such as DFT, the electronic transport properties can be calculated from the electronic structure of a material using Boltzmann transport theory. The derivation from the Boltzmann equation into the first-principles approach is based on parametrized band structure (*e.g.*, WFs) and scattering mechanism (*e.g.*, relaxation time). For a detailed explanation, see Scheidemantel *et al.*^43^ This can be done using the direct band interpolation method, as implemented in the BoltzTrap code, which was used by Ricci *et al.*.^10^ However, this technique requires the use of a highly dense *k*-mesh, which is costly. An alternative approach to the direct interpolation method is to use Wannier functions, as implemented in the BoltzWann code. This approach uses MLWFs to interpolate the band structure of the material and, with a scattering mechanism, to calculate the electrical conductivity. It has the advantage of using a less dense *k*-mesh while maintaining the accuracy of the calculation in comparison to the direct interpolation method. For a computational time and convergence comparison between the two approaches, see SI, Section 2.3.4.

In this work, the BoltzWann code using MLWFs is used to calculate electrical conductivity properties of selected materials. In our scope of work, the thermal conductivity is not investigated. The indirect interpolation using MLWFs, as implemented in BoltzWann, is as follows:4
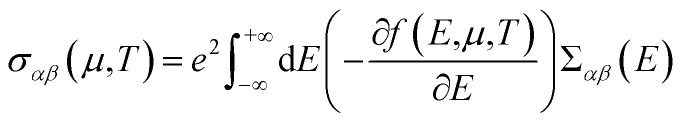
where *µ* represents the chemical potential (which is set to be around Fermi energy), *α* and *β* are the Cartesian indices, *f*(*E*, *µ*, *T*) is the Fermi–Dirac distribution function, and *Σ*_*αβ*_(*E*) is the transport distribution function (TDF). The Fermi–Dirac function represents the number of charge carriers, while the TDF represents their mobility. As such, the TDF includes an explicit sum of the group band velocities *v*_*α*,*β*_(*n*, *k*) over all quantum numbers of the system, the energy of the bands *E*_*n*,*k*_ and the relaxation time *τ*_*nk*_, as shown in eqn (5).5
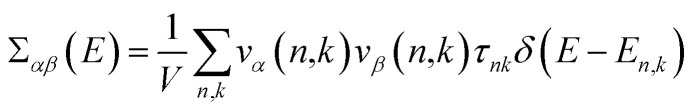


In eqn (5), *δ* is the Dirac delta (*δ* = 1 when *E* = *E*_*n*,*k*_, otherwise 0). *τ*_*nk*_ is, on the other hand, a critical parameter, affecting *Σ*_*αβ*_(*E*) and therefore *σ*_*αβ*_(*µ*, *T*) with a linear dependency. This parameter has to be specified in the input file before the calculation. The effects of the relaxation time on the electron-phonon interaction were investigated for semiconductors and compared to experimental results for anisotropic bulk structures by Allen *et al*.^44^ In previous works,^10^ a constant relaxation time of 10 fs was used. Here, we used the same relaxation time for the sake of comparability. An investigation of the effect of the relaxation time on selected systems will be carried out in future work.

The electrical conductivity *σ*_*αβ*_(*µ*, *T*) depends on the chemical potential and temperature. In a first-principles calculation, the chemical potential must be specified. Its value varies with doping or temperature. To obtain the conductivity at a given temperature, we set the chemical potential to a range that covers both the valence band maximum (VBM) and conduction band minimum (CBM). For metals, the temperature-dependent Fermi level values are found using Fermi–Dirac smearing *via* QE for each temperature. For insulators, the Fermi level is approximately in the middle of the gap and shifts with increasing temperature (see ref. 45, Section 28). In the current work, no doped systems were considered.

### 2.4 Method validation

We first validated our method before the calculations were done, see SI, Section 1. The band structures obtained after Wannierization *versus* the bands obtained directly from QE are shown in Fig. S1–S8, showing good agreement. The high-frequency permittivity values obtained using the Wannier charge center method were compared with the Berry phase method, see Fig. S9–S11, showing that our result closely follows the reference data. We validated our workflow for electrical conductivity calculation by comparing with results from MP, see Fig. S12–S14, showing a good agreement given the difference in methodology.

After successfully validating our MLWF-based framework, we can now confidently calculate the material properties and evaluate the effect of different PP libraries and structural effects. We note that in this work, PZ, PBE, and PBE + *U*_AFLOW_ DFAs are used for all calculations; benchmarking of other DFAs will be carried out in further work.

## 3 Results

As discussed in the introduction, the effect of the inconsistency between the DFA used to parametrize the PPs and that used for the calculations is to be assessed. This comparison aims to show whether an inconsistency between PP parametrization and the XC functional used can affect materials properties such as band gap and conductivity. In the inconsistency calculations, we use the PBE DFA in combination with various pslibrary (PS) PPs, which were parametrized using the BP, PBEsol, and PBE functionals, and are provided in both PAW and ultrasoft (US) forms. The results for ZnO and V_2_O_5_ are shown in Section 2.1 of the SI. For the band structures of the two materials (Fig. S15–S18), the use of inconsistent PPs can lead to ∼0.1 eV difference in the band gaps, consistent with previous work.^8^ In the extreme case of the already underpredicted band gap of ZnO, the use of the PS-US PPs with PZ-parametrization can cause a 0.3 eV difference (*i.e.*, a 40% deviation). For the V_2_O_5_ case, the effect is reduced (to below 7%) as the band gap is larger at ∼1.6 eV. The results are analogous for the electrical conductivity (Fig. S19–S22). There is no significant deviation for the V_2_O_5_ case, with the activation energies around 0.01 eV across functionals. On the other hand, there is a non-negligible effect for ZnO, especially when the PZ-parametization is used (a 0.06 eV or a 30% deviation in the activation energy from PBE and BP results). Our current results are therefore consistent with the work of Petousis *et al.*^8^ the effect of the XC functional – PP parametization inconsistency cannot be neglected for materials with a band gap below ∼1 eV; in most other cases the effect is negligible.

The effect of the atomic structure on the computed material properties, such as electrical conductivity, also has to be assessed. This was investigated for insulating materials, including the magnetic Cr_2_O_3_, using different functionals (PZ, PBE, PBE + *U*_AFLOW_) using Ps-Dojo PPs by comparing relaxed/optimized computational structure from MP (solid lines) *versus* the experimental structure from the Crystallography Open Database (dashed lines), see the Fig. S23–S28. The experimental structure provides a better agreement with the literature values of activation energy of intrinsic electrical conductivity for all materials except ZnO, discussed below in Section 3.3. In the case of MoO_3_, shown in Fig. S24, the improved agreement when the experimental structure is used is quite striking.

### 3.1 Band gaps

**Fig. 1:**
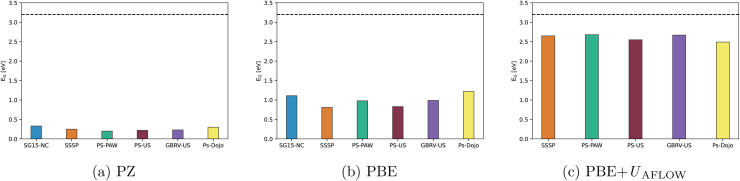
Band gap comparison for Cr_2_O_3_ across different PPs with various functionals: (a) PZ, (b) PBE, (c) PBE + *U*_AFLOW_. Experimental data (dashed line) from Abdullah *et al.*^46^

Fig. 1 shows the effect of various PPs on the computed band gaps. The band gap values are shown only for the semiconducting materials in our set, since metals do not have any band gap near the Fermi level. The available experimental values of *E*_G_ are indicated by the dashed lines in Fig. 1. For all calculations, the computed band gaps are underestimated compared to the experimental values. This underestimation is expected since the PZ and PBE DFAs are well-known for predicting lower band gaps than experimental values; as expected, the use of AFLOW + *U* values improves the agreement with experiment somewhat.

The Fig. 1 shows the typical, systematic dependence of *E*_G_ of Cr_2_O_3_ on the DFA, while PP dependence remains relatively minor. For PZ (Fig. 1(a)), the band gaps are underestimated across all PPs (∼0.2–0.4 eV), which is expected due to the well-known over-delocalization error of LDA. Changing the functional to PBE (Fig. 1(b)) provides the typical improvement of GGA over LDA across all PPs (∼0.8–1.2 eV), but still far from the experimental value (∼3.2 eV (ref. 46)). In contrast to PBE, for Cr_2_O_3_, the Hubbard correction using AFLOW + *U* values significantly improves the band gap prediction with respect to the experimental value. Since Cr_2_O_3_ is an antiferromagnetic oxide, all calculations are performed within a spin-polarized framework. Cr_2_O_3_ serves as an important stress test for our MLWFs-based framework, as it extends the material scope to include magnetic cases where spin polarization and localized *d* states play a crucial role. The results for the rest of the materials can be found in the SI, Fig. S29–S34.

The influence of different PP libraries on the band gap is minor. For example, MoO_3_ (see Fig. S29) shows nearly the same values of *E*_G_ among all PPs for PZ, PBE, and PBE + *U*_AFLOW_. The largest relative deviation is obtained for the ZnO case when PZ is used (see Fig. S33(a)), with around 40% deviation between PS-PAW and SG15-NC or PS-US, as the predicted band gap is very low (0.515 eV on average). With PBE, the deviation between PPs drops to 14% as the *E*_G_ values increase. Note that the Hubbard correction improves the band gap prediction in ZnO only slightly, to 1.25 eV on average (*i.e.*, still a 2 eV underprediction), and decreases the deviation between PPs to 7%. Finally, despite the PS-PAW and PS-US libraries being generated from the same atomic data,^17^ the band gaps obtained using these two libraries can differ. No consistent trend can be observed between the different PPs in this set of materials. However, the band gap values are largely robust concerning the choice of PPs for the selected materials.

### 3.2 High-frequency permittivity

The structural effect on predicting high-frequency permittivity was investigated in SI, Section 1.3, for different atomic structures, such as conventional and primitive non-relaxed cells, as well as primitive relaxed cells. Fig. S10 and S11 show that perfect agreement between the Wannier function and Berry phase methods can be obtained when one uses a relaxed primitive cell to predict high-frequency permittivity. The reason for that can be explained by poorer convergence of Wannierization at structures further from the energy minimum, see Tables S4 and S5.

In the following, we use primitive cells obtained from MP, *i.e.*, not relaxed cells, which leads to a discrepancy between the Wannier function and Berry phase results. The aim of this study is not to optimize structural parameters but to compare the performance of the different PP libraries and/or functionals under identical, experimentally relevant conditions. Additionally, a geometry optimization using a hybrid functional may be too costly for routine work.

**Fig. 2:**
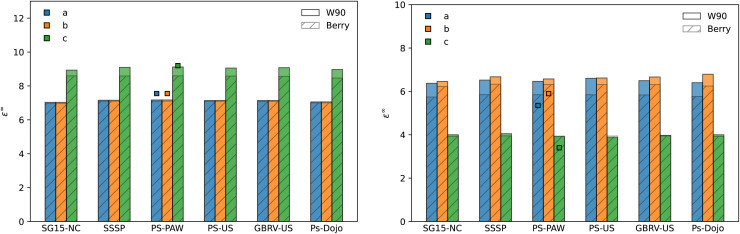
High-frequency permittivities (*ε*^*∞*^) computed from MLWF centers (solid bars) and the Berry phase (hatched bars) for TiO_2_-R (left) and V_2_O_5_ (right). Six pseudopotential libraries are compared. The squares above the PS-PAW data are taken from ref. 8. Colors represent the lattice vectors of the materials: *a* (blue), *b* (orange), and *c* (green).

Fig. 2 shows the variation of *ε*^*∞*^ for the two most interesting cases, TiO_2_-R and V_2_O_5_; the results for the rest of the materials can be found in the SI, Fig. S34–S38. The colors indicate the direction of the applied electric field as well as the lattice vector (*a*, *b*, *c*). As mentioned in SI, Section S1.3, the electric field is always applied parallel to the studied axis, *i.e.*, the Kronecker delta *δ*_*αβ*_ is always 1. All the calculations were done by using a finite external field (
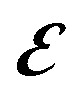
 = 0.001 Ry a.u).

For TiO_2_-R, with a tetragonal lattice, all of the computed *ε*^*∞*^ values along the *a*- and *b*-axis are identical due to the isotropic ab plane, while the *c*-axis values are different, but only slightly (see Fig. 2 (left)). The effect of PPs on Berry phase results is negligible, with the a and b components in the 6.95–7.10 range and the c component in the 8.56–8.59 range across all six PPs. For this nearly isotropic case, the differences between the MLWF-based results (solid bars) and Berry phase data (hatched bars) are minor. The agreement between the two methods is within 1%, which shows that MLWFs can reproduce Berry phase polarization very reliably. With both methods, SG15-NC gives slightly smaller values for both lattice directions by 0.1 compared to the rest.

With both methods, the calculated permittivities are lower than the MP reference values, which are 7.55 for the *a* and *b* components, and 9.20 for the c component. The reason for the deviation may be that the reference values are calculated using a relaxed primitive cell as well as a *k*-point mesh that is almost five times denser than in our work (*ρ*_k_ = 4.13*a*_0_^−3^ for MP, 0.83*a*_0_^−3^ in our work).

By contrast, V_2_O_5_ has an orthorhombic structure with three different axes and a significant anisotropy. As shown in Fig. 2 (right), with the MLWF approach, the a-axis permittivity ranges from about 6.37 (SG15-NC) to 6.60 (PS-PAW), the *b*-axis changes between 6.45 (SG15-NC) and 6.66 (GBRV-US), and the *c*-axis is between 3.8 and 4.0 across all PPs. The Berry phase calculations yield lower values, narrower spreads between PPs, but the same trends as the MLWF results.

The largest deviation for V_2_O_5_ between the MLWF and the Berry phase methods appears along the *a*-axis, with around a 10% difference. The deviation is moderate in the *b*-axis (3%) and negligible in the *c*-axis. The structure used for V_2_O_5_ in our work is a primitive cell based on the experimental structure, with a volume of 175.71 Å^3^. By contrast, the relaxed cell used to obtain the reference MP values (*a* = 5.36, *b* = 5.90, *c* = 3.40) has a volume of 196.78 Å^3^. This means the experimental structure is far from the PBE equilibrium structure, likely due to van der Waals effects. If we used the relaxed cell from the MP calculation, there would be no deviation between the reference data, the MLWF, and the Berry phase results, see Fig. S11 in the SI. It is worth noting that the MP values are calculated with almost five times denser *k*-point mesh density than our current work (3.41 *a*_0_^−3^ and 0.71 *a*_0_^−3^, respectively).

In conclusion, the Berry phase values for high-frequency dielectric permittivity can be reproduced using the Wannier function approach. Importantly, the two approaches differ in the representation of the electronic polarization: the Berry phase approach evaluates the phase of occupied states in reciprocal space, while the Wannier function method expresses the polarization using localized orbitals as charge centers in real space. Nevertheless, they give similar results for high-frequency dielectric permittivity using experimental structures, and identical results with relaxed primitive cells. The other main finding is that the choice of the PPs does not play an important role in high-frequency dielectric permittivity calculations.

### 3.3 Electrical conductivity

After validation of the MLWF-based band structures and obtaining a good agreement with the available literature data (see SI, Section S1.4),^10^ we calculated the electrical conductivity for the whole dataset, including materials with a metallic character (MoO_2_ and V_2_O_3_) and the magnetic Cr_2_O_3_ treated with spin-polarization. The temperature ranges used in the calculations are determined from the available experimental studies, since the purpose of this work is to compare with experimental values. In all cases, the experimental data were obtained from the existing experimental literature,^47–54^ focusing on the high temperature range, where the intrinsic electronic conduction mechanism ought to be dominant. Among of the reference literature works, TiO_2_-R, MoO_3_, and MoO_2_ are from single crystal samples, while the rest are polycrystalline (ZnO), annealed films (V_2_O_5_), nanoporous (TiO_2_-A), or powder (Cr_2_O_3_) samples. Note that our calculations of electrical conductivity are performed using constant relaxation time *τ*_*nk*_ = 10 fs.

**Fig. 3:**
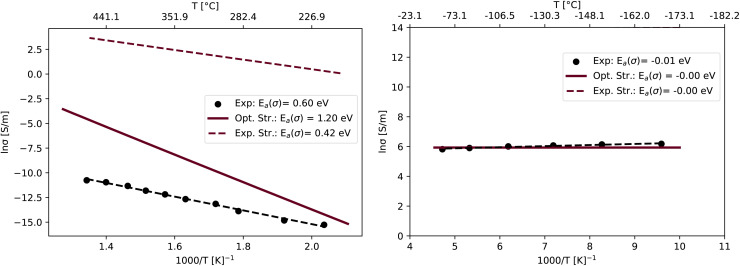
Electrical conductivity (*σ*) with PBE and PS-Dojo PPs as a function of temperature for MoO_3_ (left) and MoO_2_ (right). Calculations using optimized atomic structure (solid lines) compared to experimental atomic structure (dashed lines) and experimental data (black dots) from ref. 47 for MoO_3_, and ref. 53 for MoO_2_.

The results of the conductivity for molybdenum oxides (MoO_2_ and MoO_3_) are shown in Fig. 3, while the results using PZ and PBE + *U*_AFLOW_ can be found in the SI, Fig. S40 and S41. The effect of the PPs library on the conductivity prediction of both molybdenum oxides is negligible, see Fig. S40 and S41. This means that our workflow can predict the electrical conductivity for insulators (such as MoO_3_) and for metallic cases (such as MoO_2_). A notable difference, however, arises from the choice of atomic structure, as shown in Fig. 3 (left). For MoO_3_, the optimized structure significantly overestimates the activation energy (∼1.20 eV) compared to the experimental value (∼0.60 eV), while the experimental structure (dashed lines) estimates more accurately (∼0.42 eV). On the other hand, MoO_2_ shows almost perfect agreement with experimental values, regardless of the structure used, even though the temperature is extremely low, see Fig. 3 (right). As aforementioned, metallic materials do not have a band gap around the Fermi level; therefore, their electrons are free to move from the valence to the conduction band without thermal activation. Thus, the activation energy for metals is expected to be around zero. Note that in both cases, the experimental conductivity values are obtained using single crystals.

**Fig. 4:**
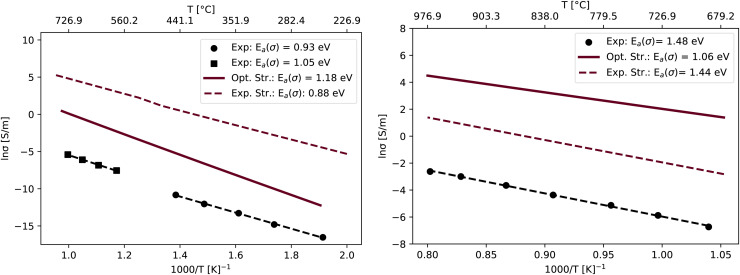
Electrical conductivity (*σ*) with PBE and PS-Dojo PPs as a function of temperature for TiO_2_-A (left) and TiO_2_-R (right). Legend as in Fig. 3, with experimental data from Ref. 55 and 51 for TiO_2_-A, and ref. 50 for TiO_2_-R.

The results of the conductivity for TiO_2_-A and TiO_2_-R are presented in Fig. 4. The effect of PPs on the electrical conductivity of TiO_2_-A and TiO_2_-R is negligible for the three DFAs used, see Fig. S42 and S43. TiO_2_-A is known to exhibit a large polaron formation at low temperature, see ref. 41, 56 and 57. The phase diagram of polarons in TiO_2_-A clearly shows that above 240 K the intrinsic conduction mechanism starts dominating over the polaron mechanism of conductivity, see Fig. 9 in ref. 41. Therefore, we calculated the electrical conductivity of TiO_2_-A above this threshold, in the range from 400 K to 800 K, where experimental data is also available.^51,55^Fig. 4 compares the calculated and experimental datasets, showing different activation energies. This indicates a strong sensitivity to sample-dependent effects such as microstructure, defect concentration, and experimental conditions. However, the deviation between the two experimental datasets (∼0.12 eV) is much lower than the deviation between the optimized atomic structure and experimental atomic structure (∼0.30 eV).

On the other hand, TiO_2_-R is known to exhibit a small polaron formation at low temperature.^39^ Above 400 K, the conduction mechanism is governed by band transport.^39^ We calculated the electrical conductivity values in the range of 850 K to 1250 K, as shown in Fig. 4 (right). The effect of the choice of structure is also here not negligible. The results from the experimental atomic structure calculations give more accurate conductivity behavior in this temperature range (∼1.44 eV) when compared to the experimental value (∼1.48 eV) obtained from single crystals, than when using the optimized structure (∼1.06 eV). This is similar to the MoO_3_ case. Finally, an improvement in the band gap prediction does not correlate with an improvement in the conductivity prediction for either of the titanium oxides, see Fig. S42 and S43 in the SI.

**Fig. 5:**
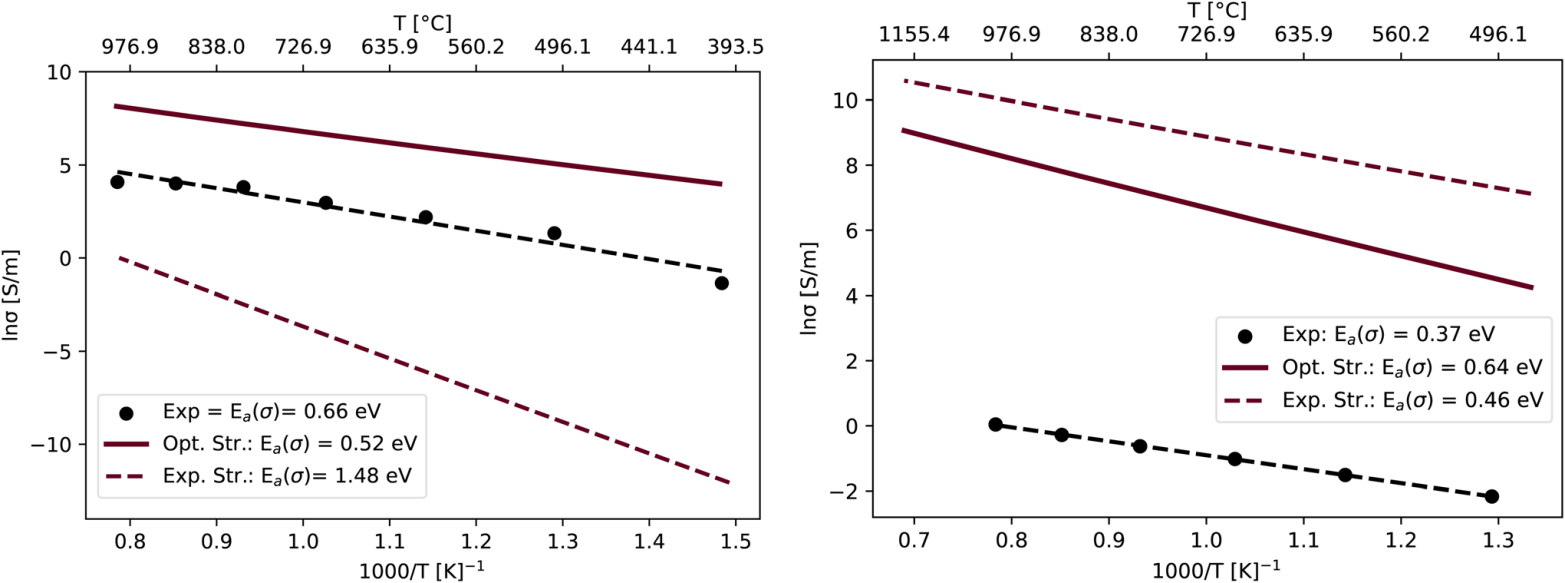
Electrical conductivity (*σ*) with PBE and PS-Dojo PPs as a function of temperature for ZnO (left) and Cr_2_O_3_ (right). Legend as in Fig. 3, with experimental data from ref. 48 for ZnO, and ref. 54 for Cr_2_O_3_.

The results of the conductivity for ZnO and Cr_2_O_3_ are shown in Fig. 5. For ZnO, despite the significantly underestimated band gap with PBE (see Fig. S33), the conductivity is captured well, with reasonable agreement in the absolute value of *σ* (within an order of magnitude), and excellent agreement in the activation energy of intrinsic conductivity *E*_a_(*σ*). The conductivity does not depend only on band gap energies (*i.e.*, the number of charge carriers), but also on band velocities (*i.e.*, charge carrier mobility), see eqn (5). Given the underprediction of the ZnO band gap and associated overprediction in the number of carriers, the charge carrier mobility here must be strongly underpredicted, leading to conductivity values consistent with experiment due to fortuitous error cancellation. This is confirmed by the use of PBE + *U*_AFLOW_, see Fig S46, which improves the band gap energy, but reduces the agreement in *E*_a_(*σ*). Additionally, ZnO exhibits an opposite sensitivity to the choice of the atomic structure compared to the other materials, as discussed in Section 3. When the experimental atomic structure is used for ZnO, a much worse agreement with experimental conductivity values is obtained, again due to the breaking of the above error cancellation. ZnO is different from the rest of the materials set, because it has a fully occupied 3d closed shell. Normally, TMOs have partially filled d-states near the Fermi level. The interaction between O 2p and Zn 3d is strong and sensitive to the bond length, which is a structural parameter.^58^ Therefore, small changes in the Zn–O distance can affect p–d repulsion. We can conclude that ZnO is very sensitive to the choice of PP, DFA, inclusion of Hubbard corrections, and the atomic structure. On the other hand, the conductivity results of Cr_2_O_3_ shown in Fig. 5 (right) confirm that our methodology can calculate the temperature dependence of the conductivity values using spin polarization, with a reasonable agreement obtained as long as the experimental atomic structure is used. Even here is the effect of the PP choice negligible, see Fig. S47.

**Fig. 6:**
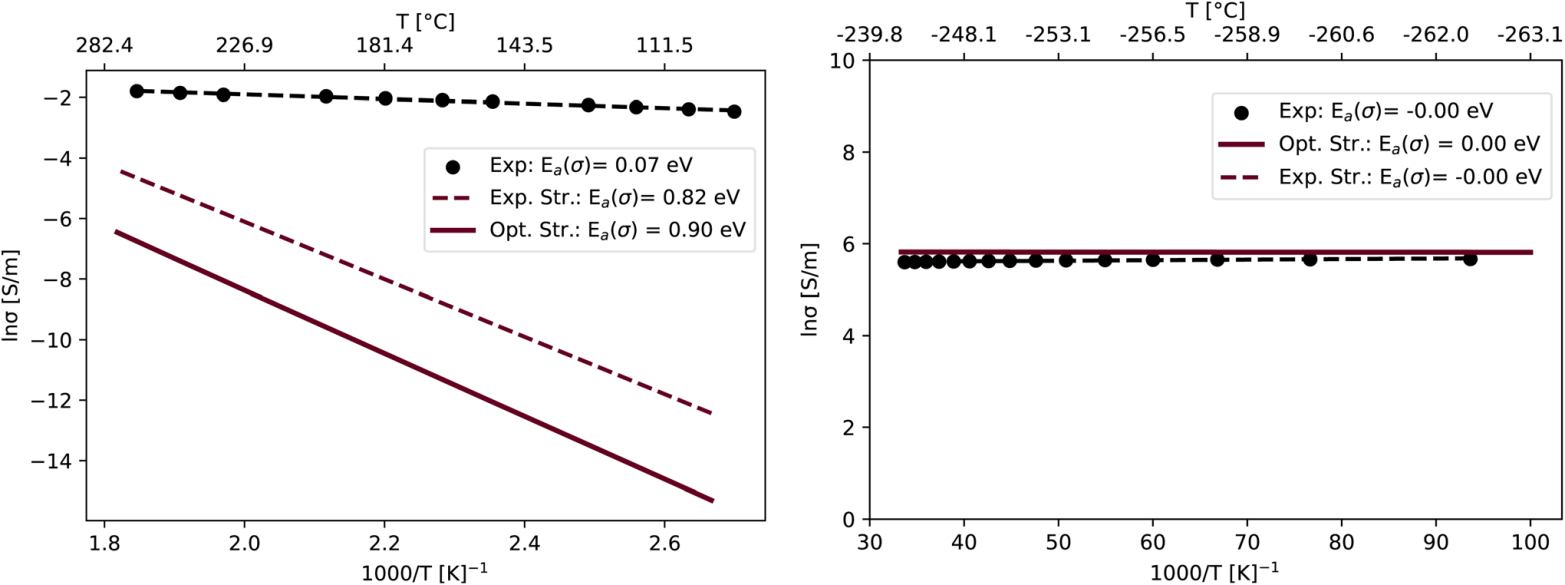
Electrical conductivity (*σ*) with PBE and PS-Dojo PPs as a function of temperature for V_2_O_5_ (left) and V_2_O_3_ (right). Legend as in Fig. 3, with experimental data from ref. 52 for V_2_O_5_, and ref. 49 for V_2_O_3_.

The results of the conductivity for V_2_O_5_ and V_2_O_3_ are presented in Fig. 6. The effect of PPs on the electrical conductivity of V_2_O_5_ and V_2_O_3_ is negligible, see Fig. S44 and S45. One can easily recognize in Fig. 6 that the prediction of the conductivity for V_2_O_5_ is in poor agreement with the existing experimental literature values. This can be explained by any of the following reasons: first, the temperature range studied may still be in the polaron regime, see Fig. 5 from Wernbacher *et al.*^59^ Therefore, the polaron hopping mechanism is contributing to the conduction. Since our model can only predict intrinsic conduction, the prediction does not show good agreement. Second, the experimental conductivity data^52^ is from an annealed film deposited on glass substrates, which is not a single crystal sample. Furthermore, V_2_O_5_ is known to form oxygen vacancies readily, even more so than titanium oxides.^60^ The effects of defects are also not modelled using our methodology. Finally, V_2_O_5_ is well-known as a layered structure, therefore the use of a dispersion-corrected DFA may be necessary to capture these effects. It is worth remembering that materials that have the highest anisotropy in our dataset also showed the highest deviation between the Berry phase approach and the MLWF approach in the high-frequency dielectric permittivity when using the experimental structures, see Fig. 2 and S38. However, the calculated activation energy of conductivity is around half of the experimental band gap,^61^ which corroborates that our methodology predicts a reasonable intrinsic conductivity. V_2_O_5_ is therefore a good candidate to investigate further, considering electron-phonon coupling effects. On the other hand, the results for the metallic V_2_O_3_ are shown in Fig. 6 (right). The absolute values of *σ* are predicted to within a factor of 1.5 and the choice of atomic structure plays no effect; the trends are therefore similar to the metallic MoO_2_ (see Fig. 3 (right)), as discussed above.

The choice of PP library therefore makes no significant difference in *σ* or *E*_a_(*σ*). However, in general, the inclusion of a Hubbard correction using the tabulated AFLOW + *U* values also does not lead to a systematic improvement in the predicted *E*_a_(*σ*). This is a common trend for all materials in the dataset. The correlation between band-gap accuracy and intrinsic electrical conductivity within the constant-relaxation-time framework is weak. On the other hand, for semiconducting materials where experimental data from single crystals is available (MoO_3_, TiO_2_-R), the use of an experimental atomic structure is necessary to obtain a good agreement with the activation energy of the intrinsic conductivity. Accurate treatment of materials such as V_2_O_5_, ZnO, and Cr_2_O_3_ may therefore require the use of an experimental structure with a more appropriate DFT approach, or a treatment beyond the constant relaxation time *τ*_*nk*_, in addition to comparing with single-crystal experimental data.

## 4 Conclusion

In this work, we tested the effect of the PPs and atomic structure on the electronic structure and electronic transport properties of materials using PZ, PBE, and PBE + *U* DFAs, including the band gap, high-frequency dielectric permittivity, and electrical conductivity. We have also assessed the feasibility of using maximally localized Wannier functions to calculate these properties. We found that the effect of PPs on the results of band gap, electrical conductivity calculations, and high-frequency dielectric permittivity is generally negligible. However, for perfect consistency between the MLWF and Berry phase approaches, the MLWFs have to be well converged, the occupied bands have to be properly disentangled, the primitive cells have to be used, and the structures should be relaxed. Moreover, we were able to calculate the temperature response of the conductivity of TMOs with both insulating and metallic character, as well as magnetic cases, allowing for material screening using a Wannier function-based workflow. We showed that the choice of atomic structure has a non-negligible impact on the electrical conductivity by comparing results computed with optimized/relaxed and experimental structures. Finally, we investigated the effect of the inconsistency between the PP parametrization and the XC-functional used, with significant impact on materials with a low calculated band gap (<1 eV), *e.g.*, ZnO. Our findings pave the road for benchmarking of density functional approximations on a wider range of materials in our future work.

## Conflicts of interest

There are no conflicts to declare.

## Supplementary Material

RA-016-D5RA09637E-s001

## Data Availability

Additional supplemental information including all QE and W90 input and output files as well as postprocessing scripts is available at DOI: https://doi.org/10.5281/zenodo.15630691. Supplementary information (SI): methodological validation and additional results for the effect of PP-XC inconsistency, atomic structure, and PP choices on the material properties such as band gap, high-frequency permittivity, and electrical conductivity as well as the computational cost comparison. See DOI: https://doi.org/10.1039/d5ra09637e.
