# Supplementary material for: Pseudopotential and structural effects on electronic properties in transition metal oxides using Wannier functions
Source: RSC Adv. 2026 Jun 5;16(33):30859–71. doi: 10.1039/d5ra09637e (PMC13245364; doi:10.1039/d5ra09637e)
Supplement: RA-016-D5RA09637E-s001 [file RA-016-D5RA09637E-s001.pdf]

Supplementary Information (SI) for RSC Advances.
This journal is © The Royal Society of Chemistry 2026

Supplementary Information (SI)

Pseudopotential and structural effects on electronic properties in transition metal oxides using Wannier functions

Aykut Öztürk*[1] and Peter Kraus†[1]

[1]Technische Universität Berlin, Conductivity and Catalysis Lab, Hardenbergstr. 40, 10623 Berlin, Germany

Additional supplemental information, including all QE and W90 input and output files as well as post-processing scripts, is available at DOI: 10.5281/zenodo.20119880.

Contents

1 Method validation **2**
- 1.1 Plane wave cut-off selection . . . 2
- 1.2 Band structure validation . . . 2
- 1.3 High-frequency permittivity validation . . . 5
- 1.4 Electrical conductivity validation . . . 9

2 Results **12**
- 2.1 Effect of PP – XC inconsistency . . . 12
  - 2.1.1 Band gaps . . . 12
  - 2.1.2 Electrical conductivity . . . 14
- 2.2 Effect of atomic structure . . . 16
- 2.3 Effect of PP library choice . . . 19
  - 2.3.1 Band gaps . . . 19
  - 2.3.2 High-frequency permittivity . . . 21
  - 2.3.3 Electrical conductivity . . . 23
  - 2.3.4 Computational cost: BoltzWann vs BoltzTrap . . . 31

*Email: oeztuerk@tu-berlin.de
†Email: peter.kraus@tu-berlin.de

1 Method validation

1.1 Plane wave cut-off selection

The cut-off selection is summarized in Table S1, listing values used for each material and pseudopotential (PP) library.

	MoO_x		TiO_2-x		V_xO_x		Cr_2O_3		ZnO		SiO_2	
	wfc	rho	wfc	rho	wfc	rho	wfc	rho	wfc	rho	wfc	rho
GBRV-US	60	480	60	480	60	480	60	480	60	480	60	480
PS-PAW	49	316	52	575	50	641	48	457	73	394	44	316
PS-US	49	316	52	575	50	641	48	457	73	394	44	316
SG15-NC	120	480	120	480	120	480	120	480	120	480	120	480
SSSP	50	400	50	400	50	400	50	400	50	400	50	400
PS-Dojo	84	336	84	336	84	336	94	336	84	376	84	336

Table S1: Plane-wave kinetic energy (ecutwfc) and charge-density (ecutrho) cut-offs used for all PP and material combinations.

	Mo	Ti	V	Zn	Cr	O
U_{AFLOW} [eV]	2.4	4.4	2.7	7.5	3.5	10^{-8}

Table S2: $+U$ parameters from AFLOW standard values [1] applied to d orbitals.

1.2 Band structure validation

The validation of the band structure is critical to ensure the consistency of the post-processing steps, such as the calculation of the electronic transport properties. We therefore validate the band structures obtained after Wannierization against those obtained directly from Quantum ESPRESSO (QE).

The results are shown in Fig. S1 for the metallic MoO_2 and in Fig. S2 for the semi-conducting MoO_3. The figures show that the Wannierization approach discussed in the Method section (see Section 2.1 in the manuscript) can be used to interpolate the Bloch wave functions, reproducing the electronic structures not only for insulators and metals but also for magnetic cases. The projected densities of states are critical to determining the orbital projections used in Wannierization. Results of validation of the electronic structures for the remaining materials can be found below, see Figures S3–S7. In all cases, the MLWF-based methodology can accurately reproduce the electronic band structures.

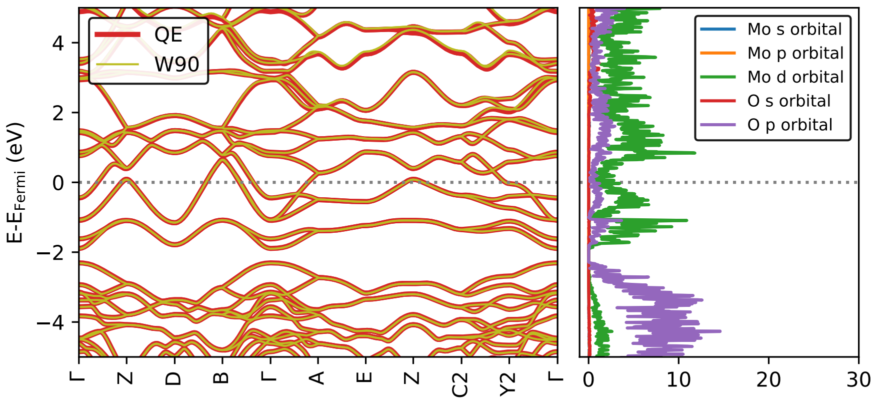

Figure S1: Validation of W90 band structures and density of states against QE data for MoO_2.

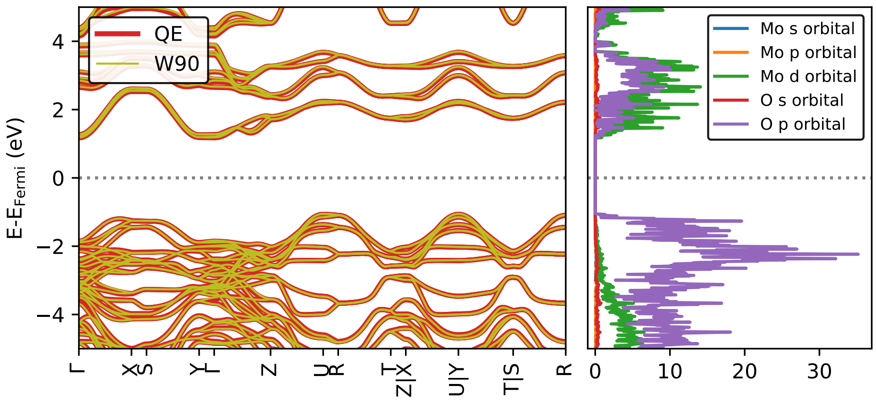

Figure S2: Validation of W90 band structures and density of states against QE data for MoO_3.

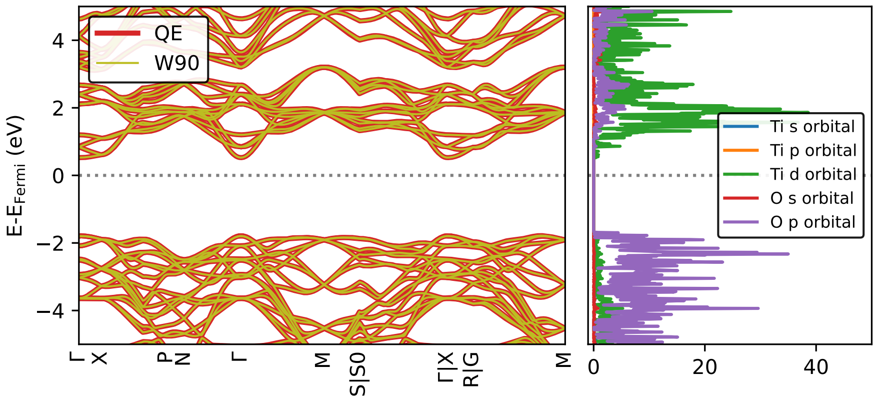

Figure S3: Validation of W90 band structures and density of states against QE data for TiO_2-A.

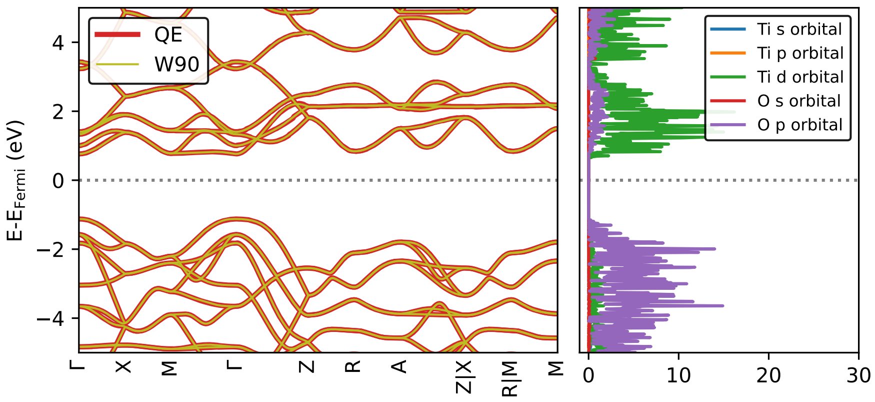

Figure S4: Validation of W90 band structures and density of states against QE data for TiO_2-R.

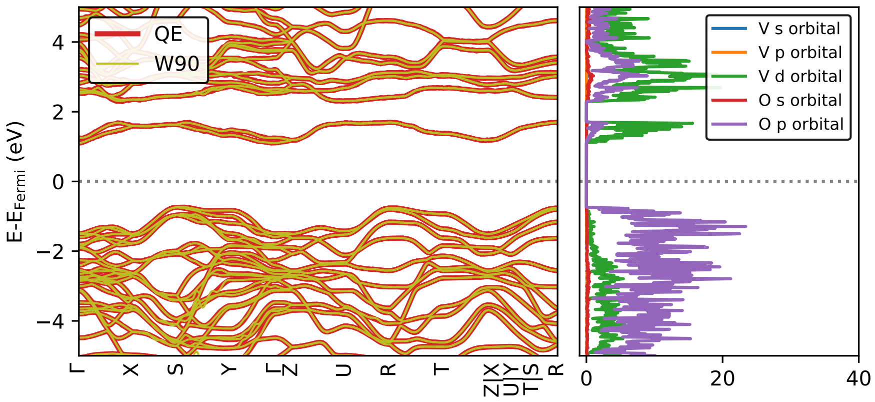

Figure S5: Validation of W90 band structures and density of states against QE data for V_2O_5.

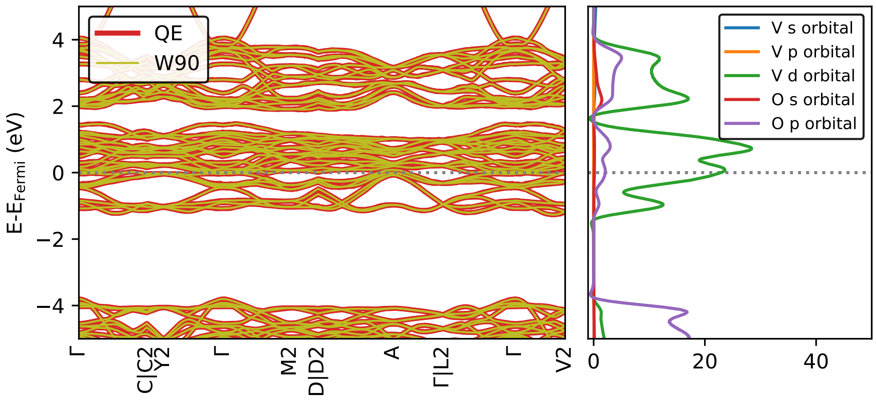

Figure S6: Validation of W90 band structures and density of states against QE data for V_2O_3.

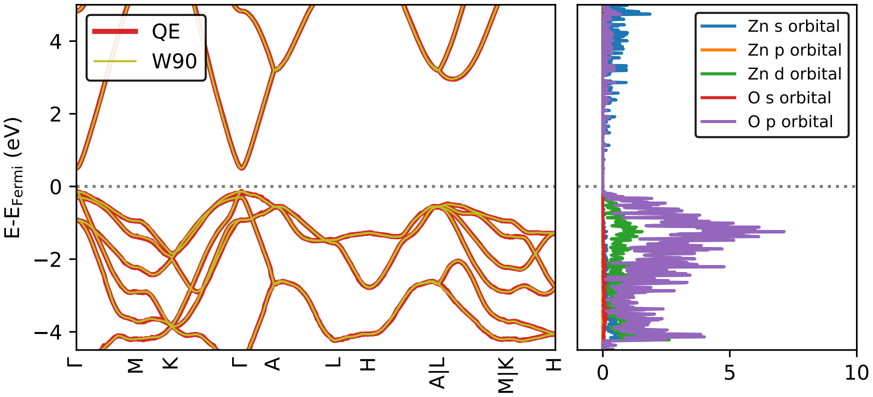

Figure S7: Validation of W90 band structures and density of states against QE data for ZnO.

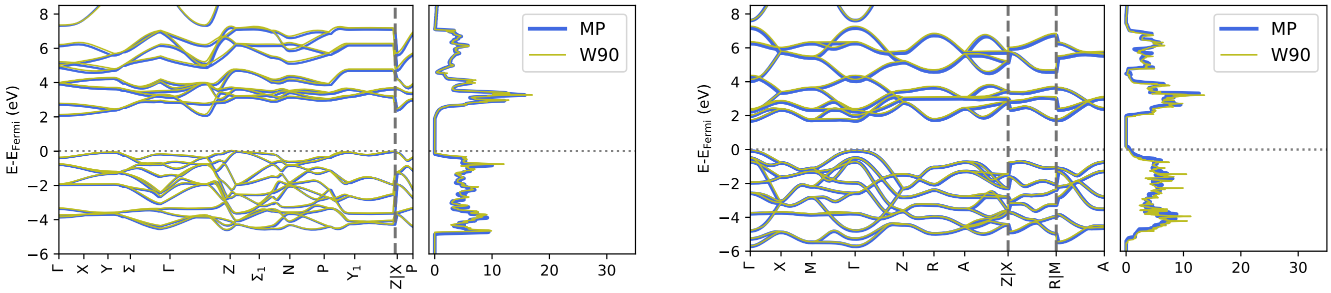

Figure S8: Validation of W90 band structures against MP data for TiO_2-A (left) and TiO_2-R (right).

As a further validation, we reproduced existing theoretical data for the TiO_2-A and TiO_2-R systems, using the results from the MP database as reference data (material IDs [mp-390] and [mp-2657] for TiO_2-A and TiO_2-R, respectively). To ensure a fair comparison, we performed our calculations using the same density functional approximation (DFA), i.e., PBE, and comparable PPs (PAW) for both materials. The results are shown in Fig. S8. The overall dispersion of valence and conduction bands is well aligned, with only slight differences in the DOS, especially in the rutile phase.

1.3 High-frequency permittivity validation

The high-frequency permittivity values were calculated using the electronic polarization when an external electric field was applied, as described through the modern theory of polarization, see Methods Section 2.3.2.

The Berry phase method follows Umari et al. [2]. The optimized wavefunctions are read from self-consistent field (SCF) calculations performed with QE (pw.x). This method calculates the electronic and ionic dipole in a.u. (atomic unit) per unit cell for each iteration, which is derived from the Berry phases of the occupied wavefunctions. The last iteration should be taken as the most converged one. This method is very well explained and implemented in the QE manual.

The Wannier function method follows Vanderbilt and Resta [3]. In this method, the Wannier charge centers are used to calculate high-frequency dielectric permittivity using the wavefunctions from the SCF calculations with an applied external field carried out with QE. The main difference between the Berry phase method and Wannier functions is that in the Berry phase approach, polarization is determined from the phases of the wavefunctions, while the Wannier charge centers are determined directly from the real-space average position of the electronic charge. The only previous application of the Wannier

function method, to our best knowledge, is by Otsuka et al. [4]. They showed that the high-frequency dielectric permittivity does not change along the directions with different external electric fields. Another finding is that the linear response of the Wannier function centers under an external electric field differs along the various directions, meaning the dielectric response is anisotropic.

Metallic systems were excluded from the high-frequency permittivity calculations, as the Berry phase approach is implemented only for insulating materials with a band gap. Since the Wannier function method uses the wave functions from QE output, it is also inapplicable for permittivity calculations for metals.

From a practical perspective, the calculation of high-frequency dielectric permittivity using Wannier functions is very sensitive to the convergence of the Wannierization. The most important point is to disentangle the wavefunctions correctly, taking only the occupied states to calculate the dielectric permittivity within the external field.

The high-frequency dielectric permittivity (ε^{∞}) was validated using α-quartz due to its well-documented dielectric response. We compare our results against MP values (material ID [mp-7000]), obtained using the DFPT method, PBE DFA, and PAW PPs. A further dataset is available from Warmbier et al. [5], where ε^{∞} was also calculated using DFPT with PAW PPs, as well as using the Berry phase and NC PPs. In our work, we used the PBE DFA with PAW PPs to calculate the dielectric permittivity of α-quartz using the same structure as in MP.

The components of the dielectric permittivity tensor of α-quartz can be divided into two categories: parallel (c, $\parallel$) and perpendicular (a and b, $\perp$) (see Eq. 1 in the Manuscript). The $\parallel$ and $\perp$ notation refers to the orientation of the optical axis of the system (i.e., c in α-quartz) with respect to the applied field. Additionally, when the electric field $\mathcal{E}$ is applied to the system, the dielectric response can be determined in the same direction ($\delta_{\alpha\beta} = 1$), or in an orthogonal direction ($\delta_{\alpha\beta} = 0$) corresponding to the diagonal and off-diagonal elements of $\varepsilon^{\infty}_{\alpha\beta}$. Here, we focus only on the diagonal elements.

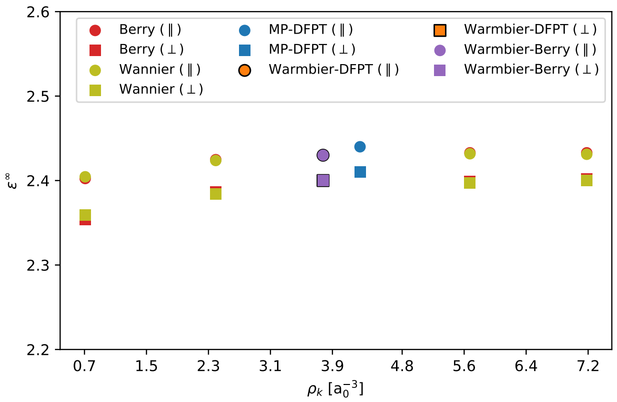

Figure S9: The high-frequency permittivity changes as a function of k-point mesh density ρ_k.

First, we investigated the effect of k-point mesh density (ρ_k) on high-frequency dielectric permittivity, with results shown in Fig. S9. We used four different k-point mesh densities. Berry phase calculations apparently converge when the k-point mesh density is $\geq$ 3.9 a_0^{-3}. The effect of the k-point mesh on the Wannier result is also examined, showing the same trend as the Berry phase, with a slightly higher k-point mesh density necessary for convergence. Note that the total energy of the system is converged even at $\rho_k = 0.71$ a_0^{-3}. The choice of the k-point mesh therefore has a non-negligible effect on dielectric permittivity, and convergence concerning ρ_k is necessary for accuracy compared to reference data. The detailed data can be found in Table S3.

Figure S9 also shows that our results (red and orange symbols) closely follow the trends in the reference data for fields applied in a parallel and perpendicular direction with respect to the optical axis. The high-frequency values computed with the Berry phase method and the Wannier function method are in close agreement, but not identical for the perpendicular direction in the non-convergence regime (i.e., $\rho_k < 5.68\ a_0^{-3}$). However, with sufficient k-point mesh density, we achieved the same result using both methods. This may be due to a convergence issue in the Wannierization, which is more sensitive than the Berry phase method.

Second, we checked the effect of the electric field strength on the dielectric permittivity. We applied three external electric field strengths: 0.0005, 0.001, and 0.002 Ry a.u. for the parallel and perpendicular cases of α-quartz. The electric field strength does not affect the dielectric permittivity of α-quartz in the range studied using either Berry phase or Wannier functions, see Table S3. One should be aware that tunneling can occur in the material if the external electric field is too high. The electric field strength should be chosen in the range below the tunneling threshold; for semiconductors, $\mathcal{E} = 0.001$ Ry a.u. is generally used [2].

Method	$\varepsilon^{\infty}(\perp\ /\ \parallel)$			ρ_k [a_0^{-3}]
	E = 0.0005 Ry a.u.	E = 0.001 Ry a.u.	E = 0.002 Ry a.u.	
Berry phase		2.35 / 2.40		0.71
		2.39 / 2.42		2.39
	2.40 / 2.43	2.40 / 2.43	2.40 / 2.43	5.68
		2.40 / 2.43		7.18
Wannier		2.36 / 2.40		0.71
		2.38 / 2.42		2.39
	2.40 / 2.43	2.40 / 2.43	2.40 / 2.43	5.68
		2.40 / 2.43		7.18
Berry phase [5]		2.40 / 2.43		3.77
DFPT [5]		2.40 / 2.43		3.77
DFPT [MP: mp-7000] [6]		2.41 / 2.44		4.26

Table S3: Comparison of high-frequency dielectric permittivity ε^{∞} of α-quartz calculated using various methods at different electric fields.

Finally, to obtain perfect agreement between the Wannier function and Berry phase methods, the structure used has to be the same, relaxed, primitive cell, as shown in Fig. S10 and S11 for the cases of V_2O_5 and TiO_2-A, respectively. The Wannier function method gives higher values in comparison with the Berry phase method for the conventional cell of TiO_2-A: around 0.8 for the ab plane, while the c-direction matches well. The results of the primitive cell calculations with Wannier and Berry phase methods are much closer, especially for the ab plane; however, the c-direction deviates by about 0.6. The same trend was observed for V_2O_5, for which the Wannier function method tends to slightly overestimate the values in comparison with the Berry phase method.

The relaxation of the cell parameters is necessary to obtain exact agreement between the Wannier function and Berry phase methods. This is due to the poorer convergence of Wannierization at structures further from the energy minimum. The convergence of the total spread of the Wannier functions is shown in

Tables S4 and S5, where one can see clearly that the relaxed structure has the lowest spread for the TiO_2-A and V_2O_5. The discrepancy observed between the Berry-phase and Wannier functions results from a convergence issue in the localization of Wannier functions. For a specific example in the TiO_2-A case, the first eight Wannier functions have a spread in the range 0.27 to 0.41 in the primitive cell, while the range is narrower between 0.31 and 0.32 for the relaxed cell. This shows that the relaxed cells remain similarly localized and become more uniform after relaxation.

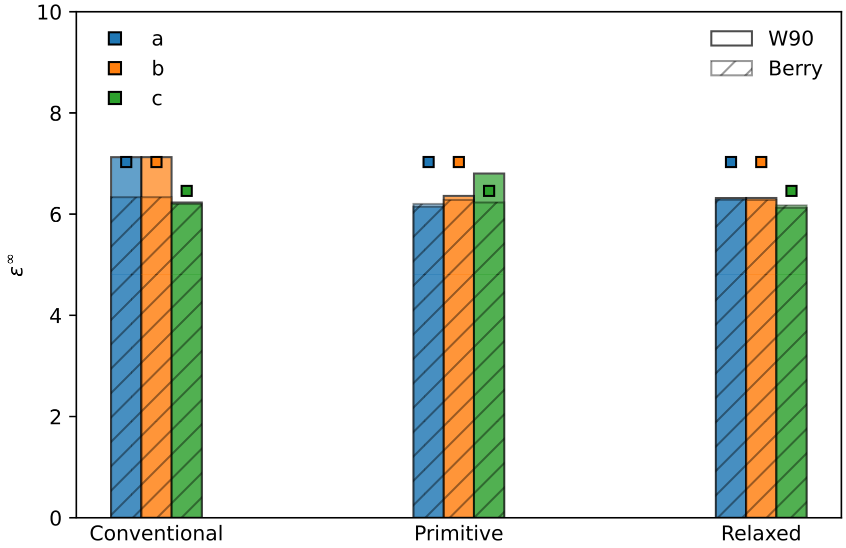

Figure S10: High-frequency permittivities (ε^{∞}) of TiO_2-A computed from MLWF centers (solid bars) and the Berry phase (hatched bars) with PS-PAW PPs. Calculations using the conventional, primitive, and relaxed unit cells. The squares above each column are taken from Ref. [7]. Colors represent the lattice parameters of the materials: a (blue), b (orange), and c (green).

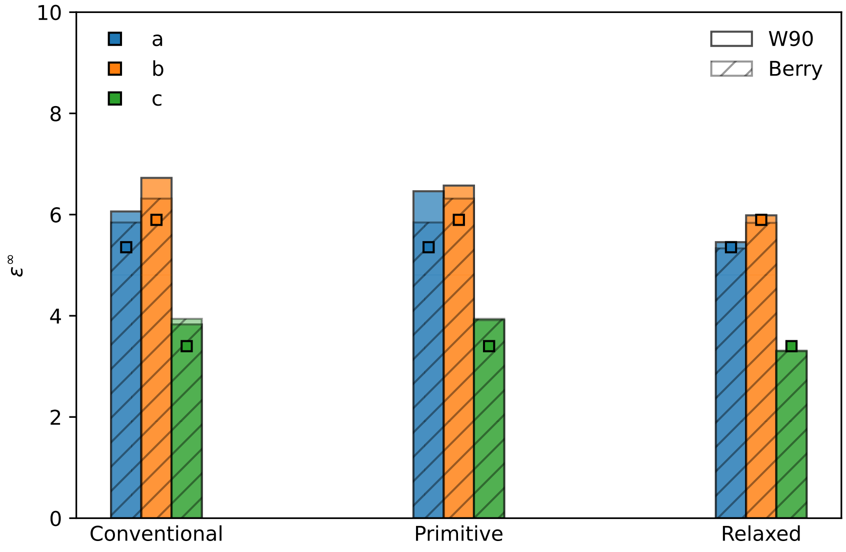

Figure S11: High-frequency permittivities (ε^∞) of V_2O_5 computed from MLWF centers (solid bars) and the Berry phase (hatched bars) with PS-PAW PPs. Calculations using the conventional, primitive, and relaxed unit cells. The squares above each column are taken from Ref. [7]. Colors represent the lattice parameters of the materials: a (blue), b (orange), and c (green).

	Conventional	Primitive	Relaxed
a [$\Delta\Omega_{\text{tot}}$]	−0.0026295	−0.0016525	−0.0001015
b [$\Delta\Omega_{\text{tot}}$]	−0.0026311	−0.0070390	−0.0008070
c [$\Delta\Omega_{\text{tot}}$]	−0.0013030	−0.0017750	−0.0000262

Table S4: Convergence of total Wannier function spreads for TiO_2-A for all three axes under an electric field.

	Conventional	Primitive	Relaxed
a [$\Delta\Omega_{\text{tot}}$]	−0.0029872	−0.0028875	−0.0035239
b [$\Delta\Omega_{\text{tot}}$]	−0.0041198	−0.0068657	−0.0049145
c [$\Delta\Omega_{\text{tot}}$]	−0.0039509	−0.0078926	−0.0002150

Table S5: Convergence of total Wannier function spreads of V_2O_5 for all three axes under an electric field.

1.4 Electrical conductivity validation

The validation of our conductivity calculation method using MLWFs is done by comparing three materials (TiO_2-A, TiO_2-R, and ZnO) with available data from Ricci et al. [8]. As discussed above, Ricci et al. used Fourier interpolation of band structure as implemented in BoltzTrap. By contrast, in our work, we use MLWFs to interpolate the band structure and calculate the transport properties of materials.

The validation calculations are done using the relaxed structures from MP (material IDs mp-2133, mp-2657, and mp-390 for TiO_2-A, TiO_2-R, and ZnO, respectively), the constant relaxation time approximation, the same DFA (PBE), and similar PP (PAW) to ensure consistency. All data retrieved from the Materials Project (MP) are from database version v2025.06.09.

Fig. S12 shows that for TiO_2-A, the absolute values are in excellent agreement with the reference data.

A good agreement with only a constant offset is obtained for TiO_2-R, see Fig. S13. For ZnO, shown in Fig. S14, the results diverge at low temperatures. This might occur due to the difference in k-point meshes used in the two studies, as in Ref. [8], a more than 20 times denser k-point mesh has been used than in our work.

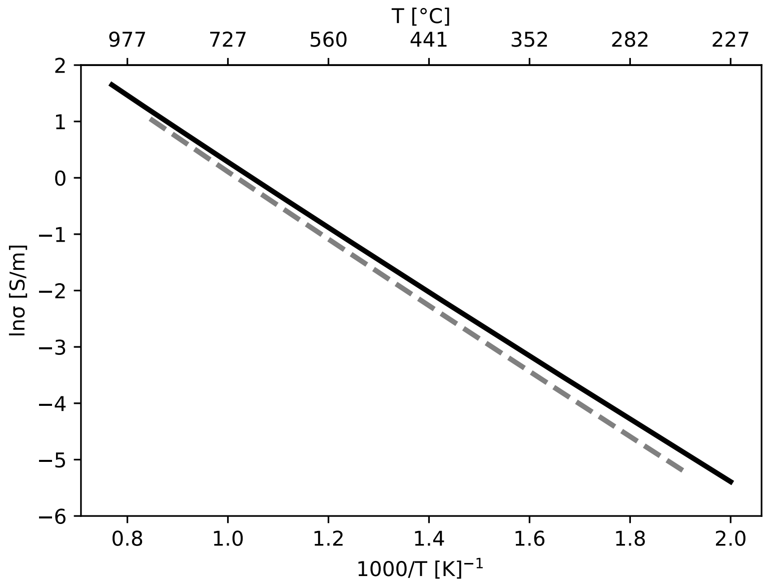

Figure S12: Validation of electrical conductivity calculations for TiO_2-A. Solid lines indicate results from current work, while dashed lines represent results from Ricci et al. [8]

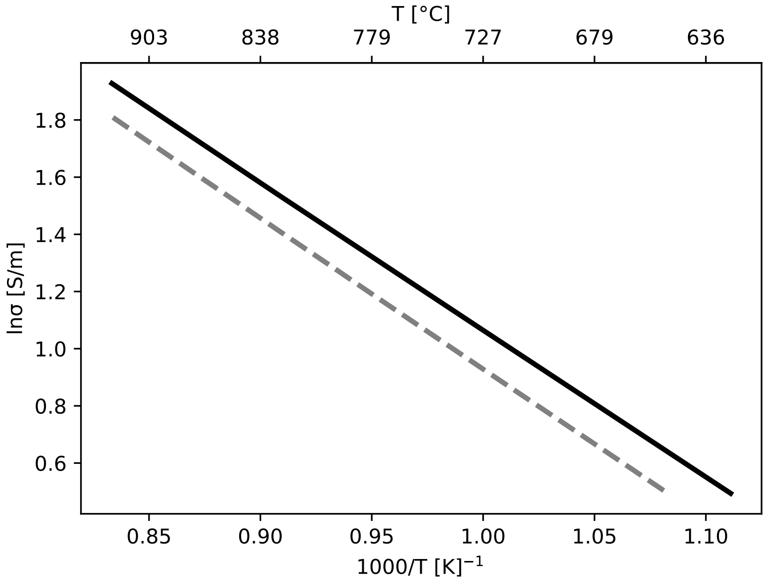

Figure S13: Validation of electrical conductivity calculations for TiO_2-R. Solid lines indicate results from current work, while dashed lines represent results from Ricci et al. [8]

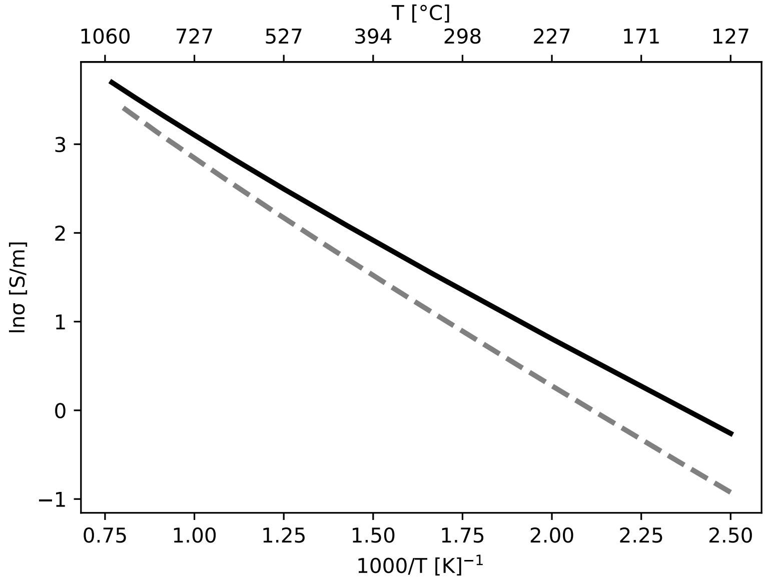

Figure S14: Validation of electrical conductivity calculations for ZnO. Solid lines indicate results from current work, while dashed lines represent results from Ricci et al. [8]

To further compare the approaches for the calculation of electronic transport, we calculated the activation energies of conductivity ($E_A(\sigma)$) from the Arrhenius-like plots shown in Figs. S12–S14. As there is a linear dependency between conductivity and relaxation time, if the relaxation time constant were higher, the line on the Arrhenius plot would shift up, but its slope would not change. Therefore, the $E_A(\sigma)$ is not affected by changes in the relaxation time constant. As shown in Table S6, the deviation between the two methods is less than 0.02 eV for all three materials. This confirms the consistency of both transport approaches for evaluating the electrical conductivity with temperature changes.

Table S6: Comparison of activation energies of conductivity $E_a(\sigma)$ with reference data. [8]

Material	$E_A(\sigma)$ (eV)	
	Current work	Ricci et al. [8]
TiO_2-A	1.17	1.05
TiO_2-R	1.05	1.02
ZnO	0.51	0.45

2 Results

2.1 Effect of PP – XC inconsistency

In this section, we investigate the inconsistency between the XC functional used in the calculations and the XC functional used to create the PPs for ZnO and V_2O_5. We use the PBE DFA with pslibrary (PS) PPs parametrized for the BP, PBEsol, PZ, and PBE DFAs, both in their PAW as well as US formulation.

2.1.1 Band gaps

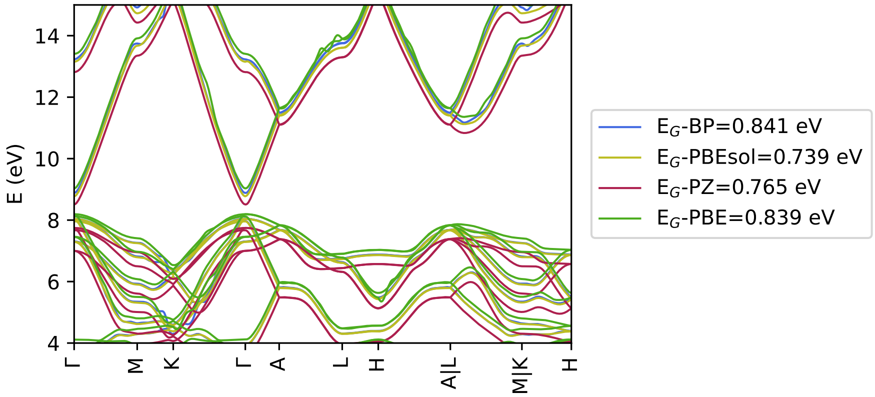

Figure S15: Band gap of ZnO using PBE and PS-PAW PPs parametrized for different functionals (BP, PBEsol, PZ, and PBE).

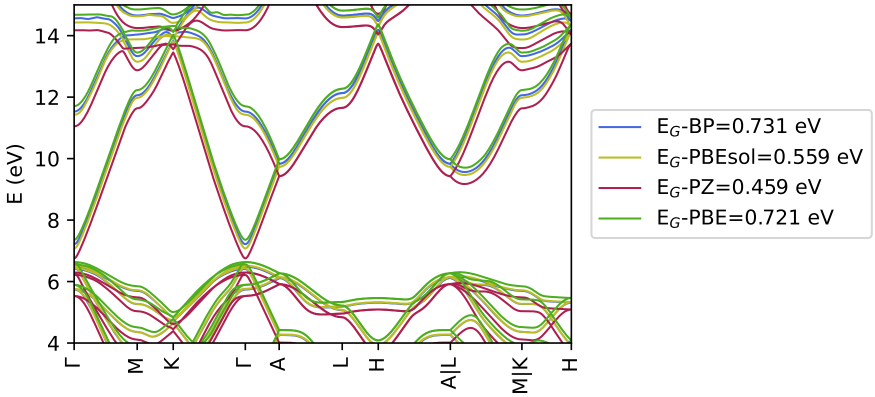

Figure S16: Band gap of ZnO using PBE and PS-US PPs parametrized for different functionals (BP, PBEsol, PZ, and PBE).

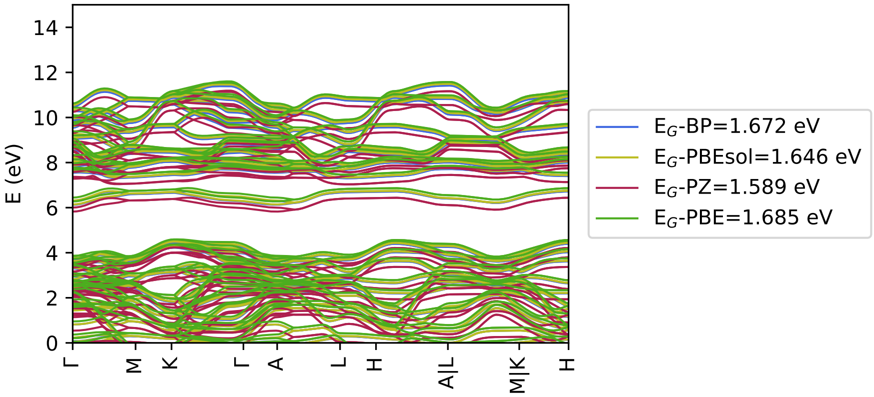

Figure S17: Band gap of V_2O_5 using PBE and PS-PAW PPs parametrized for different functionals (BP, PBEsol, PZ, and PBE).

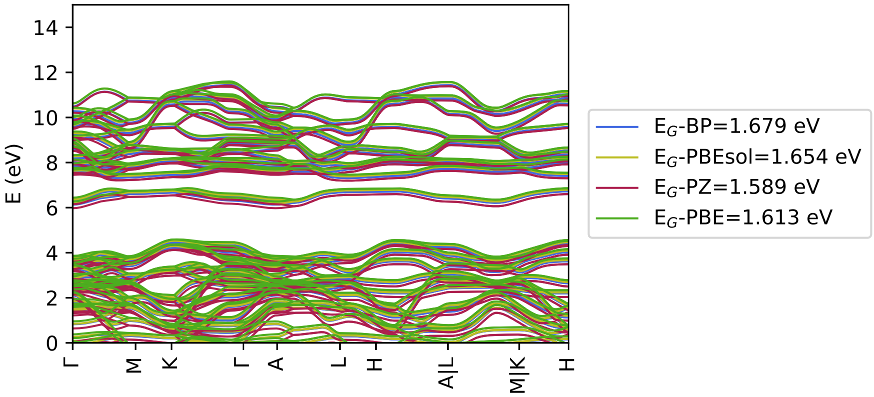

Figure S18: Band gap of V_2O_5 using PBE and PS-US PPs parametrized for different functionals (BP, PBEsol, PZ, and PBE).

2.1.2 Electrical conductivity

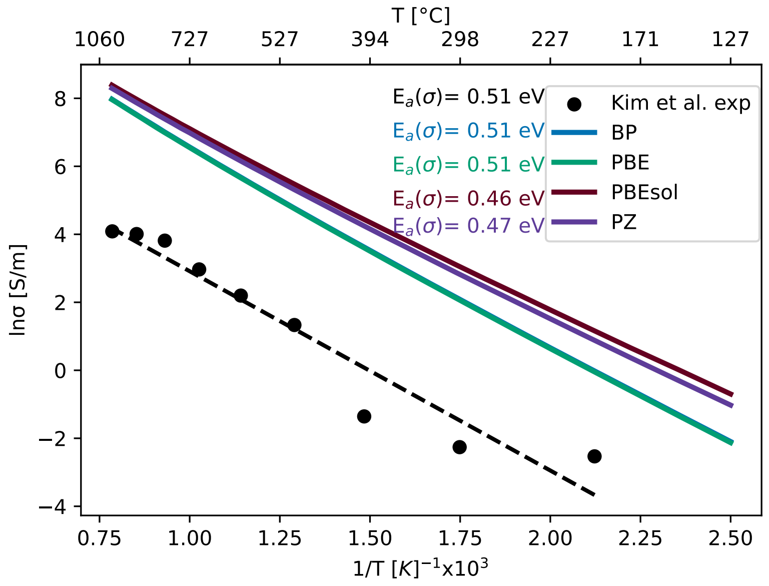

Figure S19: The electrical conductivity (σ) of ZnO vs temperature, calculated using PBE DFA and PS-PAW PPs parametrized for different functionals (BP, PBEsol, PZ, and PBE). The activation energies are shown in the legend for the functionals (solid lines) and experimental study (black dots) [Kim et al. [9]].

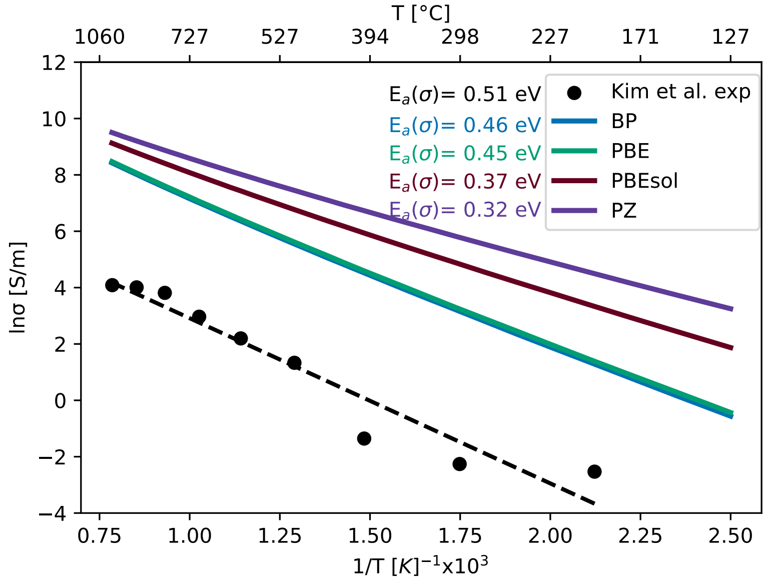

Figure S20: The electrical conductivity (σ) of ZnO vs temperature, calculated using PBE DFA and PS-US PPs parametrized for different functionals (BP, PBEsol, PZ, and PBE). The activation energies are shown in the legend for the functionals (solid lines) and experimental study (black dots) [Kim et al. [9]].

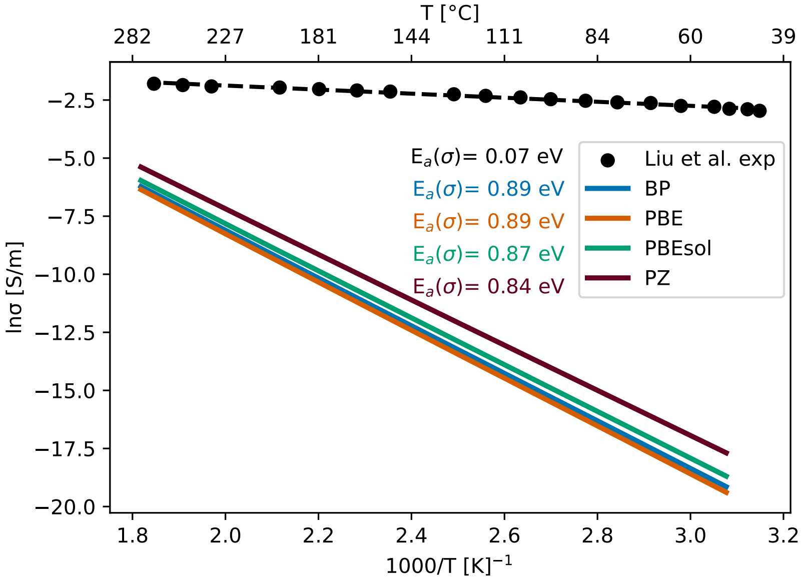

Figure S21: The electrical conductivity (σ) of V_2O_5 vs temperature, calculated using PBE DFA and PS-PAW PPs parametrized for different functionals (BP, PBEsol, PZ, and PBE). The activation energies are shown in the legend for the functionals (solid lines) and experimental study (black dots) [Liu et al. [10]].

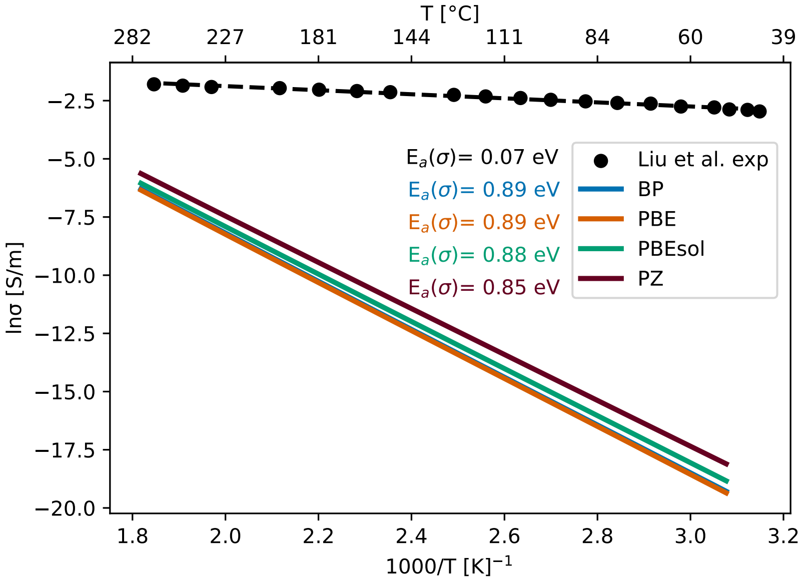

Figure S22: The electrical conductivity (σ) of V_2O_5 vs temperature, calculated using PBE DFA and PS-US PPs parametrized for different functionals (BP, PBEsol, PZ, and PBE). The activation energies are shown in the legend for the functionals (solid lines) and experimental study (black dots) [Liu et al. [10]].

2.2 Effect of atomic structure

In this part, we investigate the effect of the starting structure on electrical conductivity properties. We used two different functionals (PZ and PBE) and AFLOW $+U$ values for the Hubbard correction for PBE with PseudoDojo PP. Results with relaxed (optimized) structures from MP shown as solid lines, results with experimental structures shown as dashed lines.

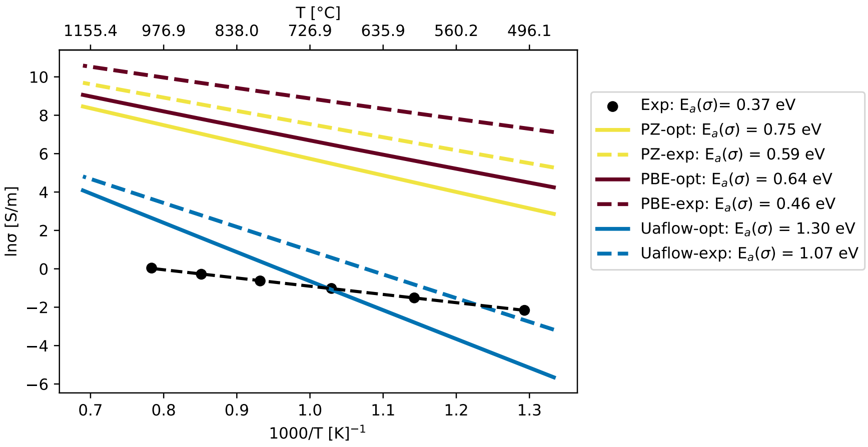

Figure S23: The electrical conductivity (σ) of Cr_2O_3 vs temperature, calculated using PZ, PBE and PBE+U_{AFLOW} DFAs with PseudoDojo PPs. The activation energies are shown in the legend for the functionals (solid and dashed lines) and experimental study (black dots) [Nagai et al. [11]].

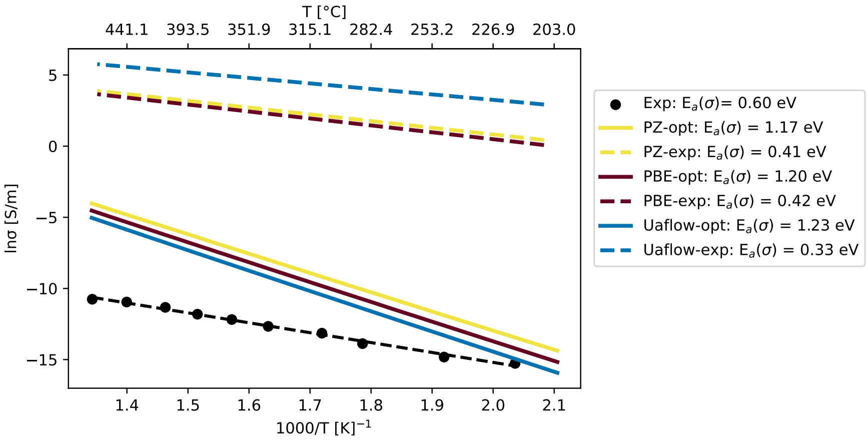

Figure S24: The electrical conductivity (σ) of MoO_3 vs temperature, calculated using PZ, PBE and PBE+U_{AFLOW} DFAs with PseudoDojo PPs. The activation energies are shown in the legend for the functionals (solid and dashed lines) and experimental study (black dots) [Deb et al. [12]].

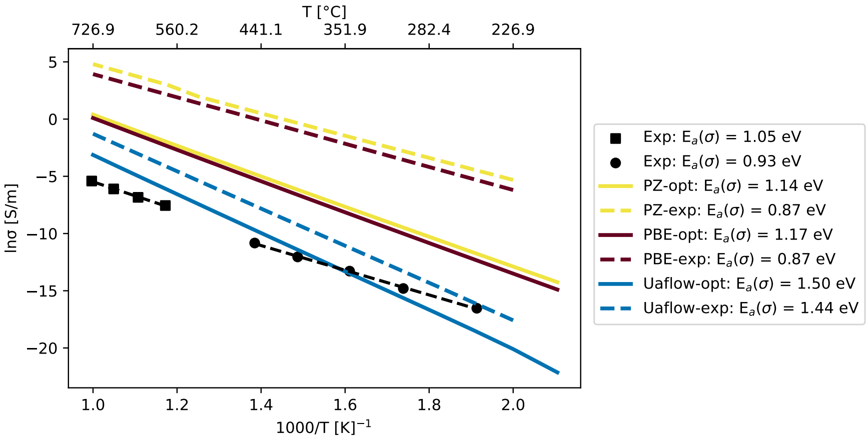

Figure S25: The electrical conductivity (σ) of TiO_2-A vs temperature, calculated using PZ, PBE and PBE+U_{AFLOW} DFAs with PseudoDojo PPs. The activation energies are shown in the legend for the functionals (solid and dashed lines) and experimental data at high temperatures (black squares) [Knauth et al. [13]], at lower temperatures (black points) [Dittrich et al. [14]].

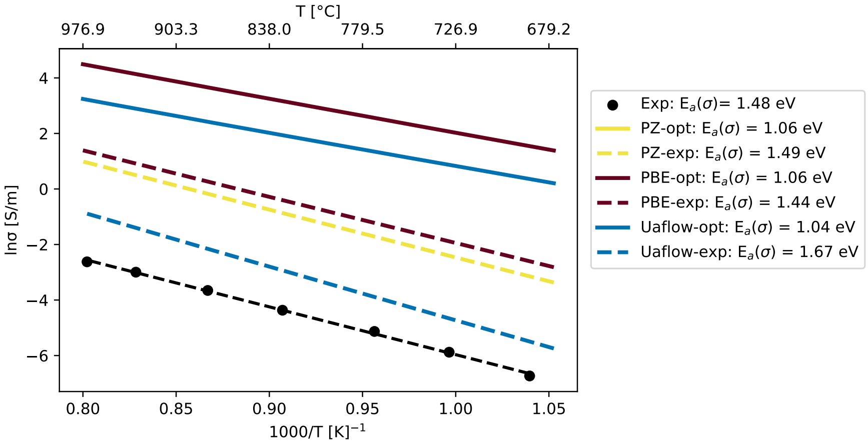

Figure S26: The electrical conductivity (σ) of TiO_2-R vs temperature, calculated using PZ, PBE and PBE+U_{AFLOW} DFAs with PseudoDojo PPs. The activation energies are shown in the legend for the functionals (solid and dashed lines) and experimental study (black dots) [Greener et al. [15]].

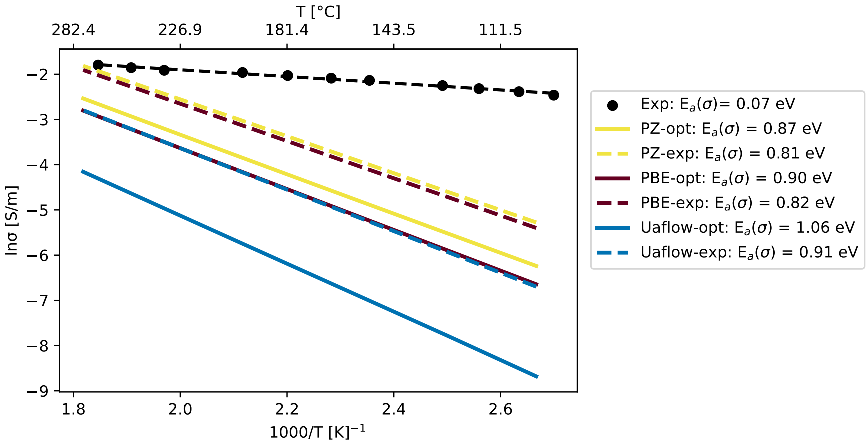

Figure S27: The electrical conductivity (σ) of V_2O_5 vs temperature, calculated using PZ, PBE and PBE+U_{AFLOW} DFAs with PseudoDojo PPs. The activation energies are shown in the legend for the functionals (solid and dashed lines) and experimental study (black dots) [Liu et al. [10]].

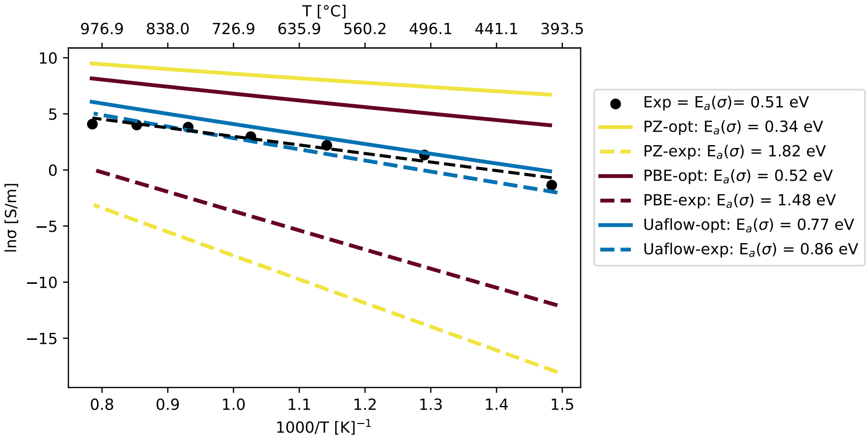

Figure S28: The electrical conductivity (σ) of ZnO vs temperature, calculated using PZ, PBE, and PBE+U_{AFLOW} DFAs with PseudoDojo PPs. The activation energies are shown in the legend for the functionals (solid and dashed lines) and experimental study (black dots) [Kim et al. [9]].

2.3 Effect of PP library choice

In this section, the calculations were done using optimized/computational structures from MP. No experimental structure was used for the PP comparison.

2.3.1 Band gaps

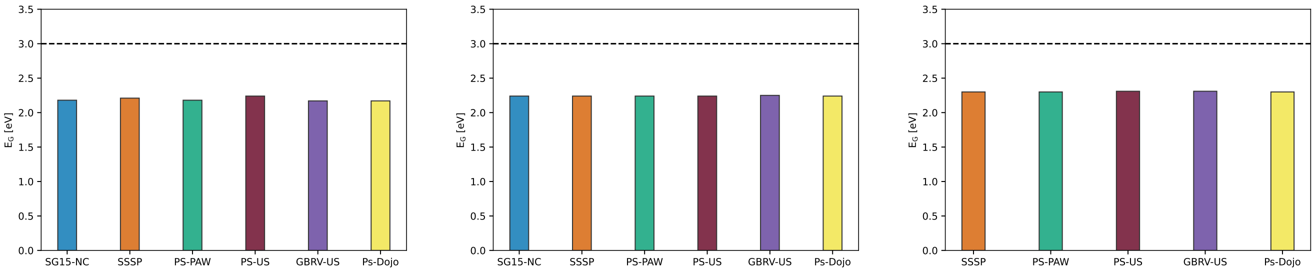

(a) PZ (b) PBE (c) PBE+U_{AFLOW}

Figure S29: Band gap comparison for MoO_3 across different PPs with the indicated DFAs. Experimental data (dashed line) from Deb et al. [12].

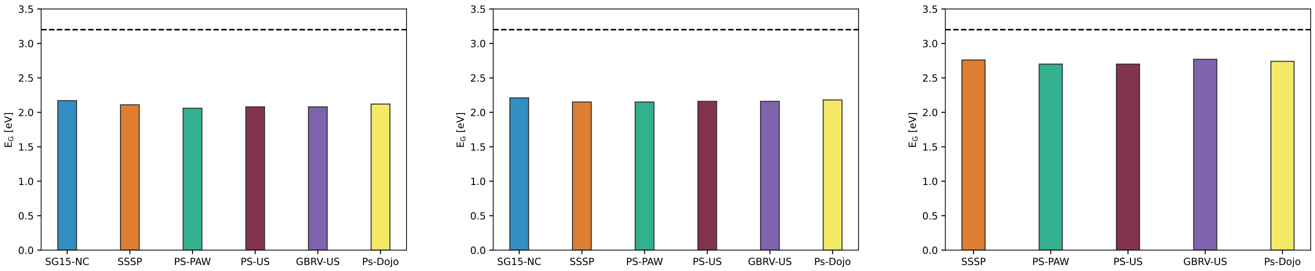

(a) PZ (b) PBE (c) PBE+U_{AFLOW}

Figure S30: Band gap comparison for TiO_2-A across different PPs with the indicated DFAs. Experimental data (dashed line) from Tang et al. [16].

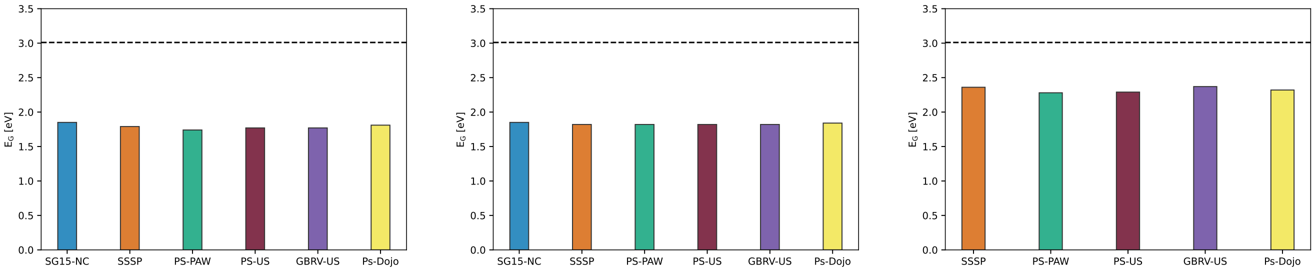

(a) PZ (b) PBE (c) PBE+U_{AFLOW}

Figure S31: Band gap comparison for TiO_2-R across different PPs with the indicated DFAs. Experimental data (dashed line) from Pascual et al. [17].

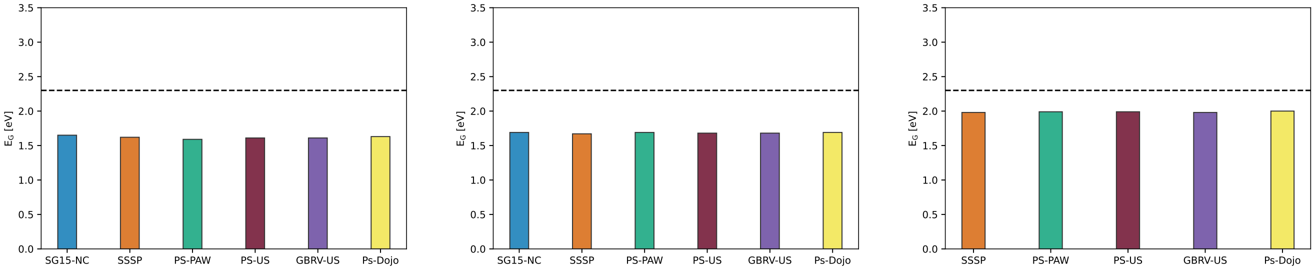

(a) PZ (b) PBE (c) PBE+U_{AFLOW}

Figure S32: Band gap comparison for V_2O_5 across different PPs with the indicated DFAs. Experimental data (dashed line) from Bodo et al. [18].

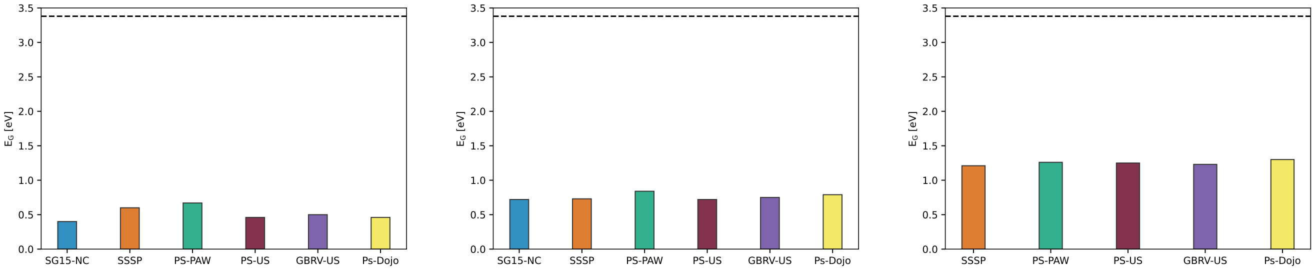

(a) PZ (b) PBE (c) PBE+U_{AFLOW}

Figure S33: Band gap comparison for ZnO across different PPs with the indicated DFAs. Experimental data (dashed line) from Liang et al. [19].

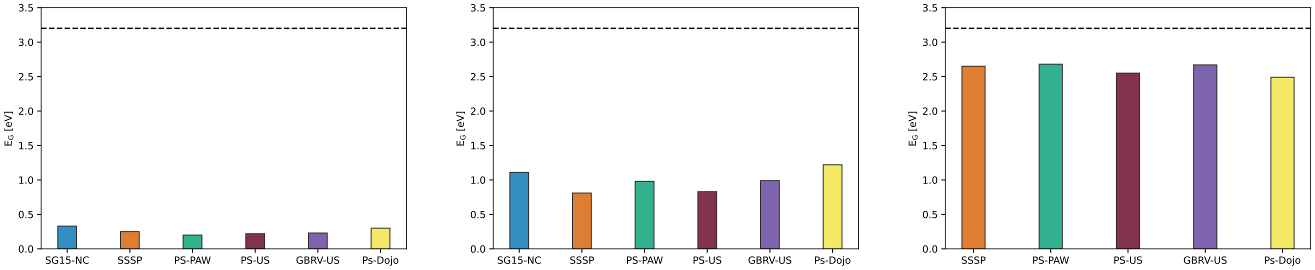

(a) PZ (b) PBE (c) PBE+U_{AFLOW}

Figure S34: Band gap comparison for Cr_2O_3 across different PPs with the indicated DFAs. Experimental data (dashed line) from Abdullah et al. [20].

2.3.2 High-frequency permittivity

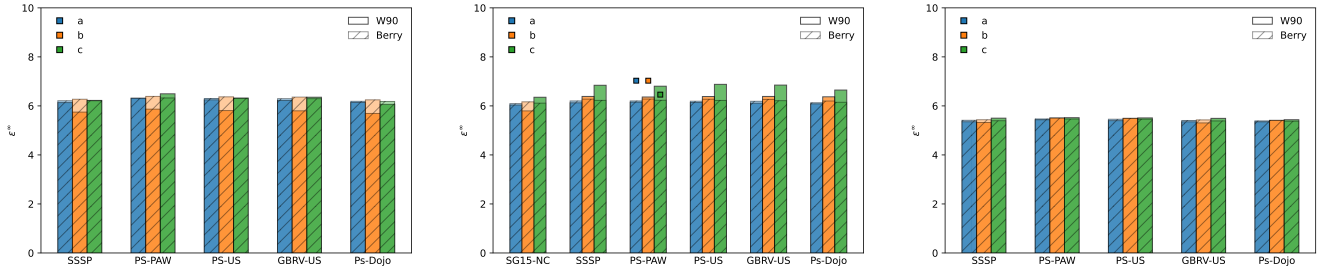

(a) PZ (b) PBE (c) PBE+U_{AFLOW}

Figure S35: High-frequency permittivities (ε^{∞}) computed from MLWF centers (solid bars) and the Berry phase (hatched bars) for TiO_2-A. The pseudopotential libraries are compared across the indicated DFAs. The three squares above the PS-PAW PP are taken from Petousis et al. [7]. Colors represent the lattice parameters of the materials: a (blue), b (orange), and c (green).

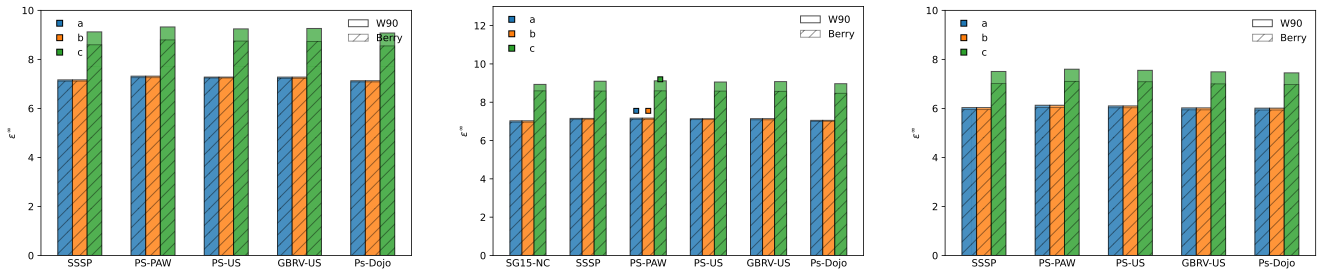

(a) PZ (b) PBE (c) PBE+U_{AFLOW}

Figure S36: High-frequency permittivities (ε^{∞}) computed from MLWF centers (solid bars) and the Berry phase (hatched bars) for TiO_2-R. The pseudopotential libraries are compared across the indicated DFAs. The three squares above the PS-PAW PP are taken from Petousis et al. [7]. Colors represent the lattice parameters of the materials: a (blue), b (orange), and c (green).

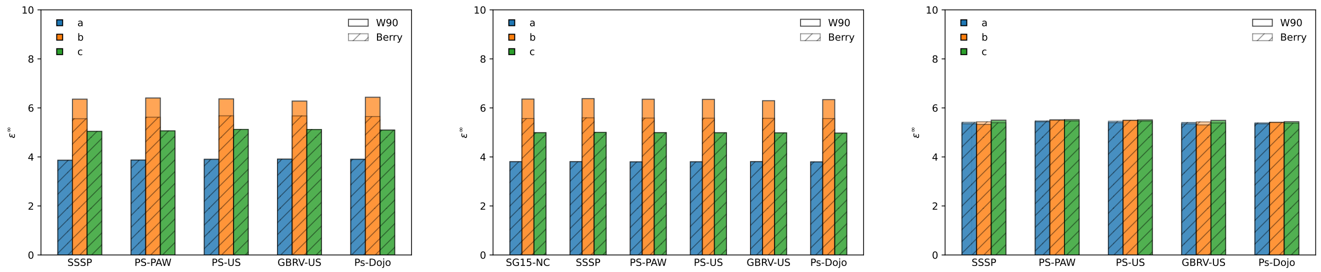

(a) PZ (b) PBE (c) PBE+U_{AFLOW}

Figure S37: High-frequency permittivities (ε^{∞}) computed from MLWF centers (solid bars) and the Berry phase (hatched bars) for MoO_3. The pseudopotential libraries are compared across the indicated DFAs. Colors represent the lattice parameters of the materials: a (blue), b (orange), and c (green).

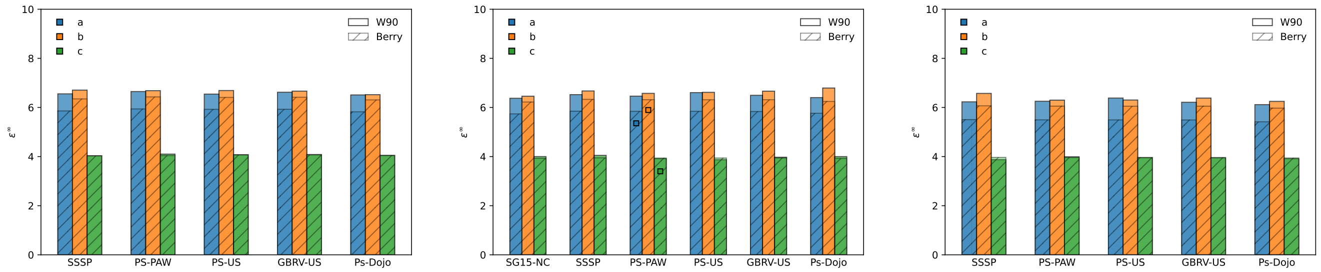

(a) PZ (b) PBE (c) PBE+U_{AFLOW}

Figure S38: High-frequency permittivities (ε^∞) computed from MLWF centers (solid bars) and the Berry phase (hatched bars) for V_2O_5. The pseudopotential libraries are compared across the indicated DFAs. The three squares above the PS-PAW PP are taken from Petousis et al. [7]. Colors represent the lattice parameters of the materials: a (blue), b (orange), and c (green).

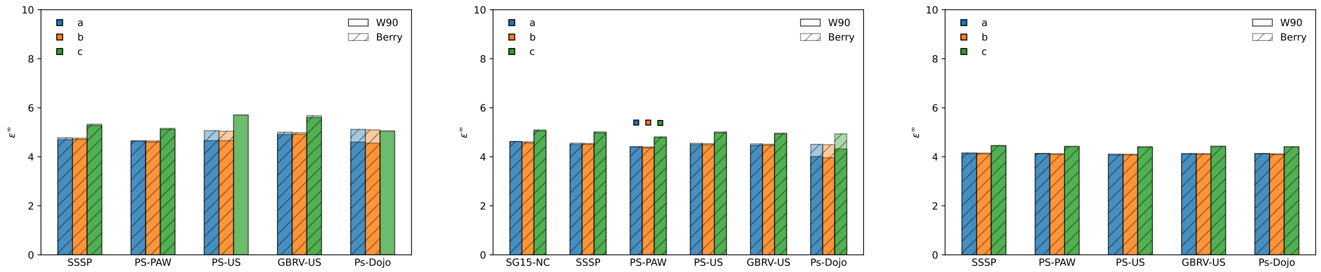

(a) PZ (b) PBE (c) PBE+U_{AFLOW}

Figure S39: High-frequency permittivities (ε^∞) computed from MLWF centers (solid bars) and the Berry phase (hatched bars) for ZnO. The pseudopotential libraries are compared across the indicated DFAs. The three squares above the PS-PAW PP are taken from Petousis et al. [7]. Colors represent the lattice parameters of the materials: a (blue), b (orange), and c (green).

2.3.3 Electrical conductivity

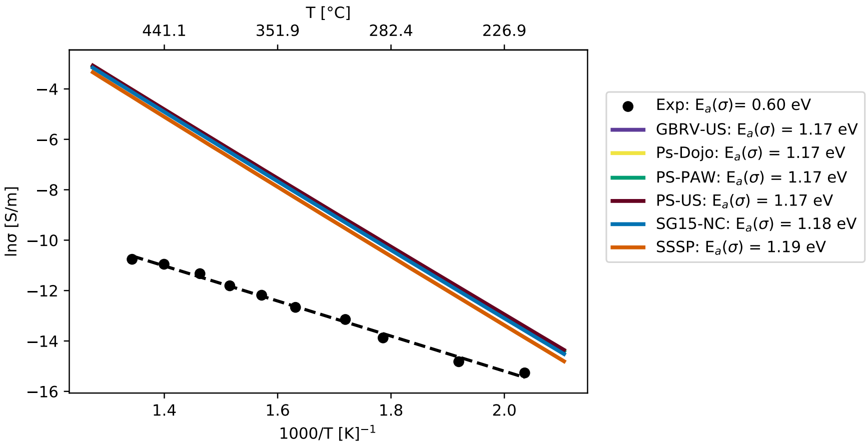

(a) PZ

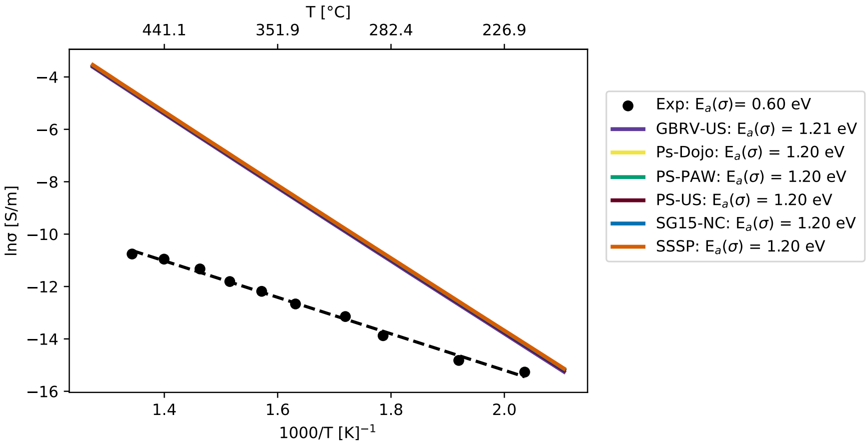

(b) PBE

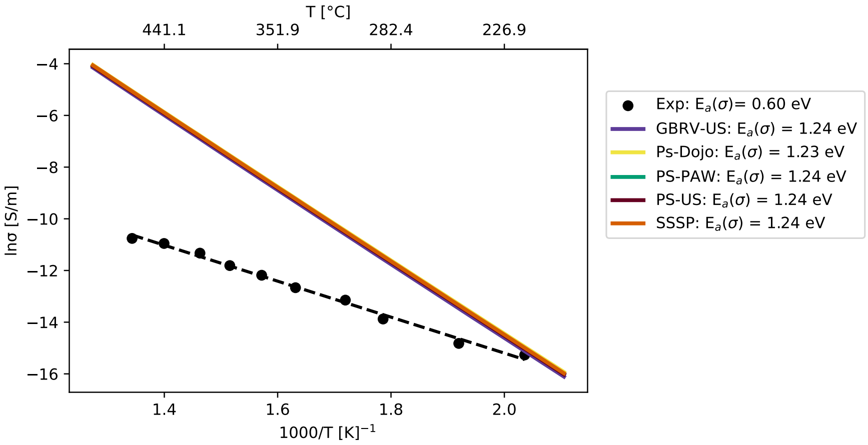

(c) PBE+U_{AFLOW}

Figure S40: The electrical conductivity (σ) of MoO_3 vs temperature. The pseudopotential libraries are compared separately for each of the indicated DFAs. The activation energies are shown in the legend for the PPs libraries (solid lines) and experimental study (black points) [Deb et al. [12]].

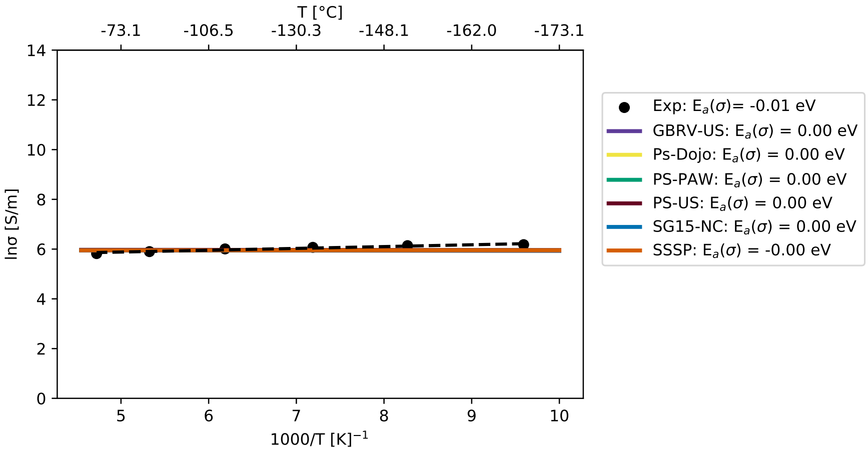

(a) PZ

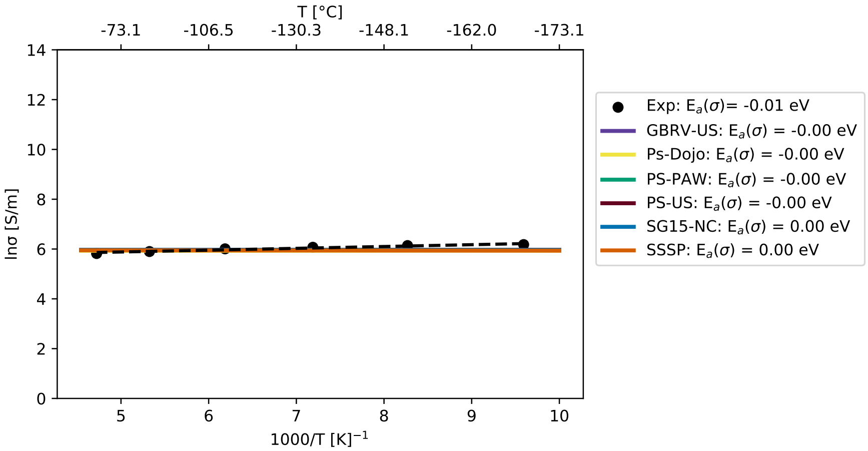

(b) PBE

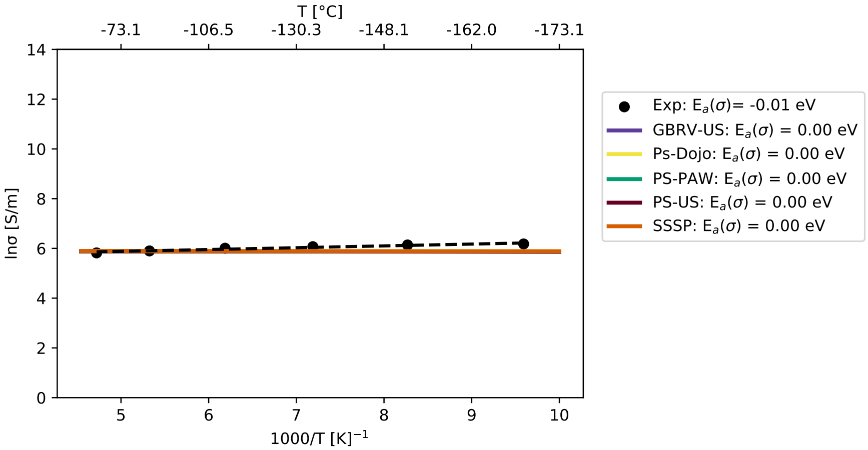

(c) PBE+U_{AFLOW}

Figure S41: The electrical conductivity (σ) of MoO_2 vs temperature. The pseudopotential libraries are compared separately for each of the indicated DFAs. The activation energies are shown in the legend for the PPs libraries (solid lines) and experimental study (black points) [Ben-Dor et al. [21]].

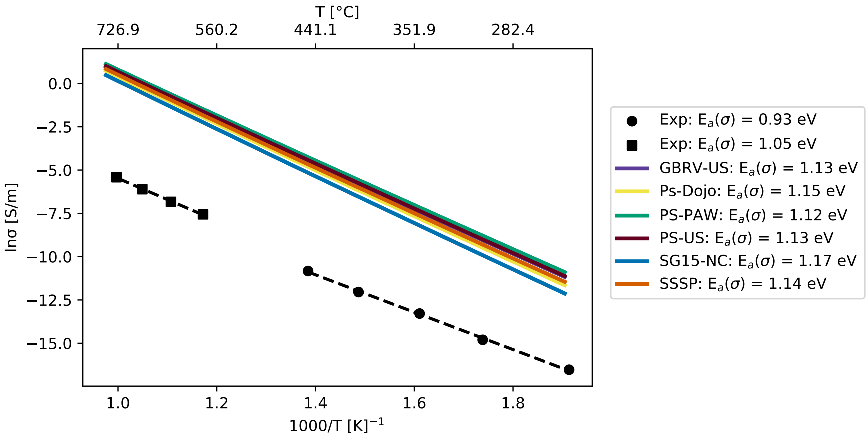

(a) PZ

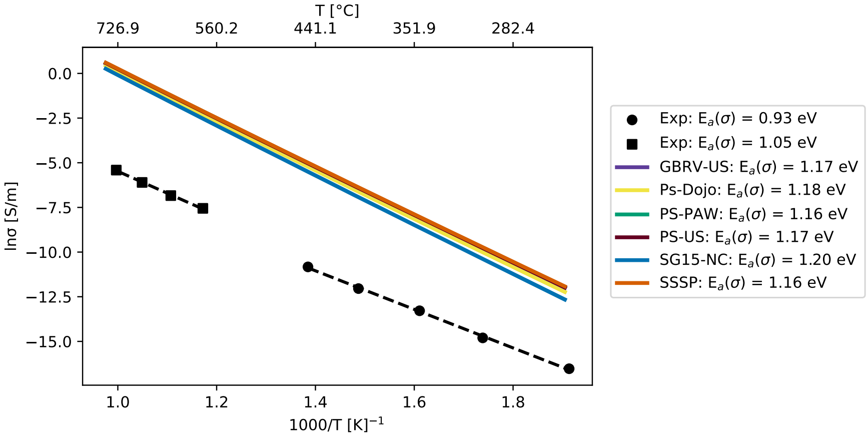

(b) PBE

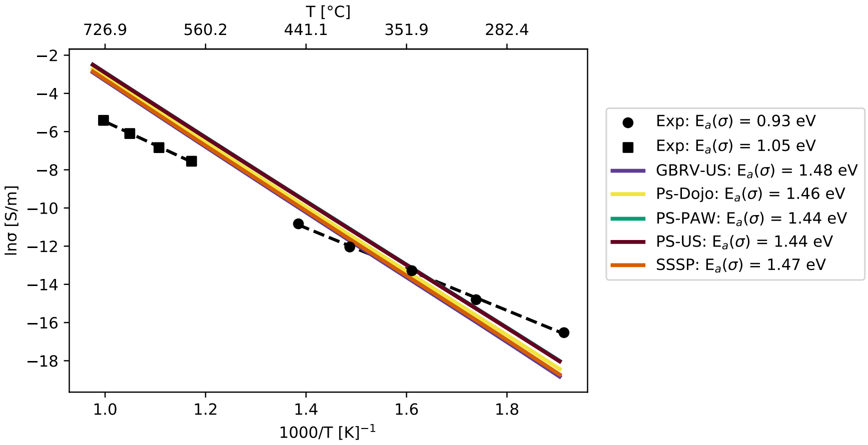

(c) PBE+U_{AFLOW}

Figure S42: The electrical conductivity (σ) of TiO_2-A vs temperature. The pseudopotential libraries are compared separately for each of the indicated DFAs. The activation energies are shown in the legend for the PPs libraries (solid lines) and experimental data at high temperatures (black squares) [Knauth et al. [13]] and at lower temperatures (black points) [Dittrich et al. [14]].

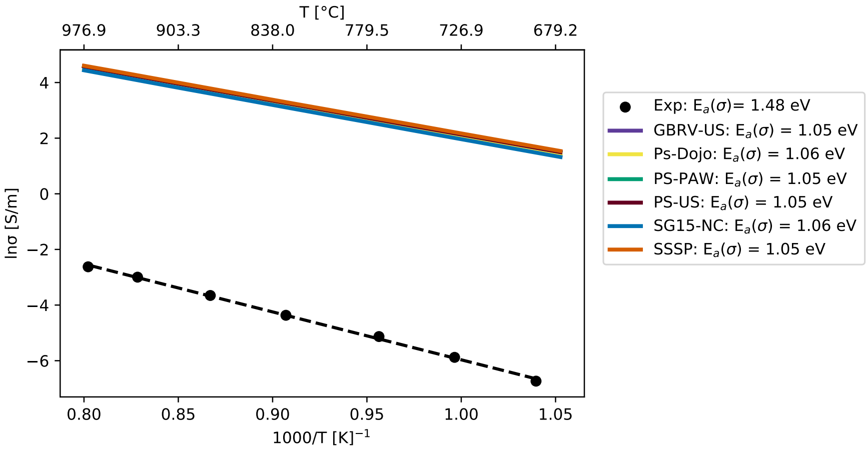

(a) PZ

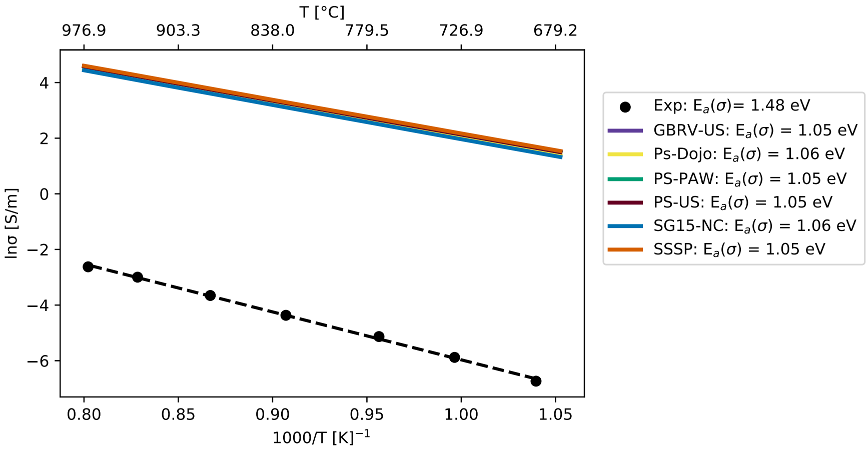

(b) PBE

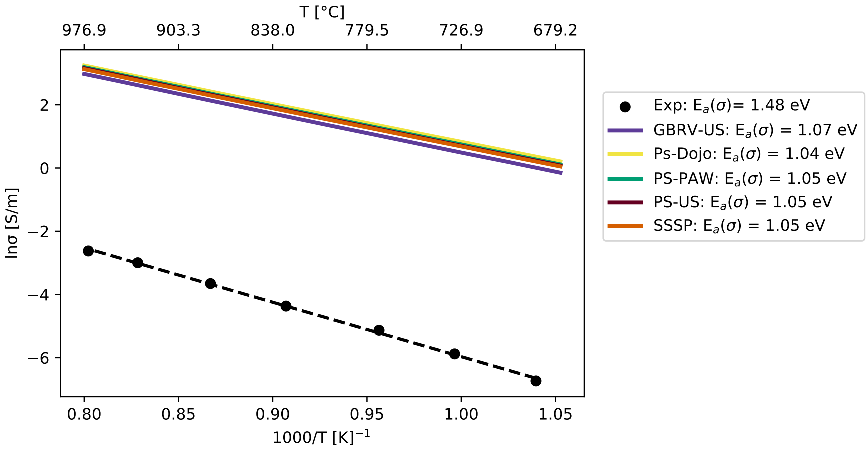

(c) PBE+U_{AFLOW}

Figure S43: The electrical conductivity (σ) of TiO_2-R vs temperature. The pseudopotential libraries are compared separately for each of the indicated DFAs. The activation energies are shown in the legend for the PPs libraries (solid lines) and experimental study (black points) [Greener et al. [15]].

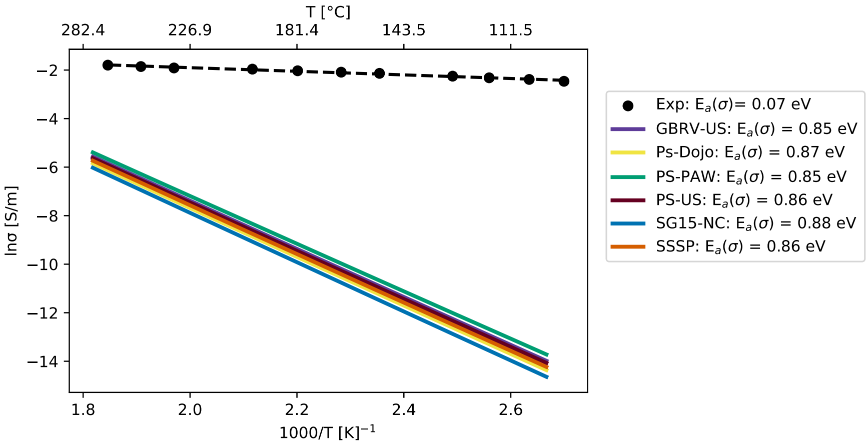

(a) PZ

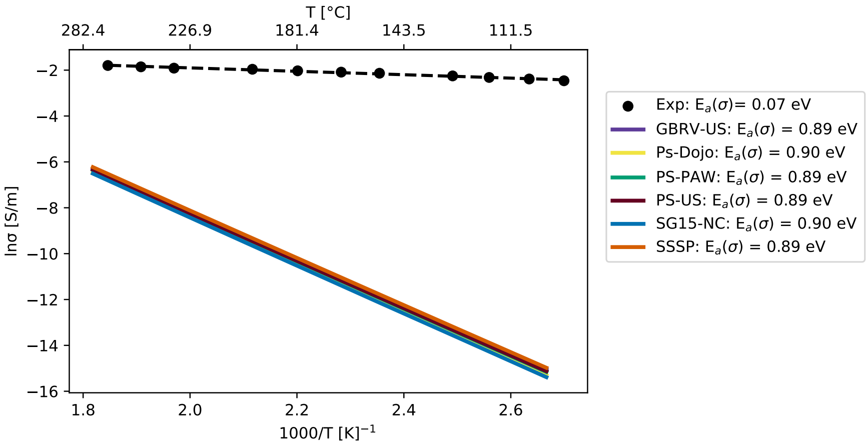

(b) PBE

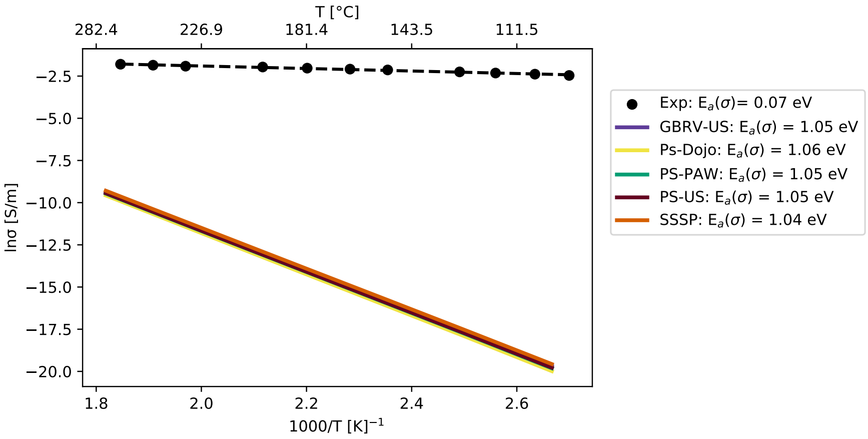

(c) PBE+U_{AFLOW}

Figure S44: The electrical conductivity (σ) of V_2O_5 vs temperature. The pseudopotential libraries are compared separately for each of the indicated DFAs. The activation energies are shown in the legend for the PPs libraries (solid lines) and experimental study (black points) [Liu et al. [10]].

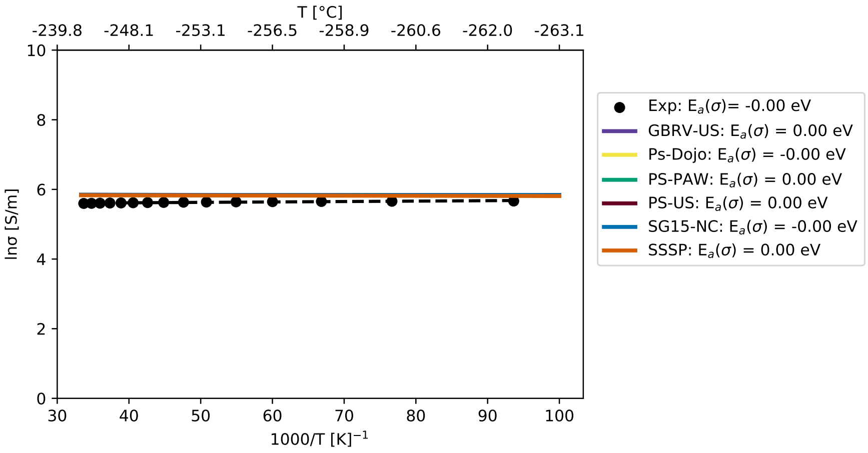

(a) PZ

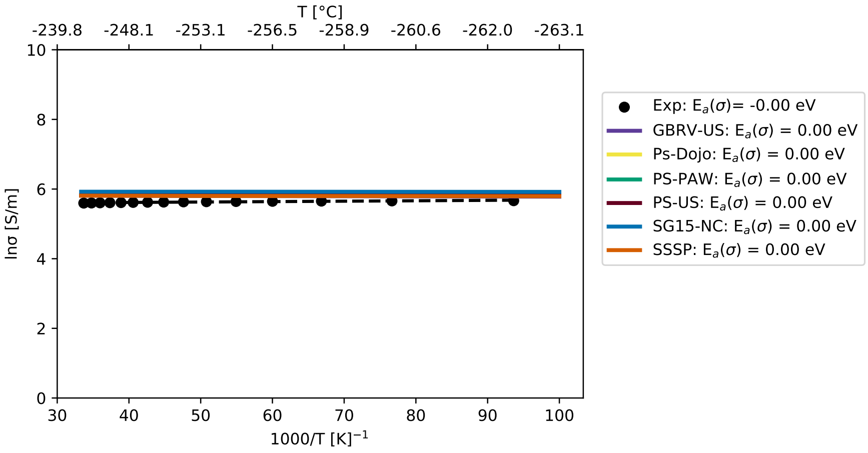

(b) PBE

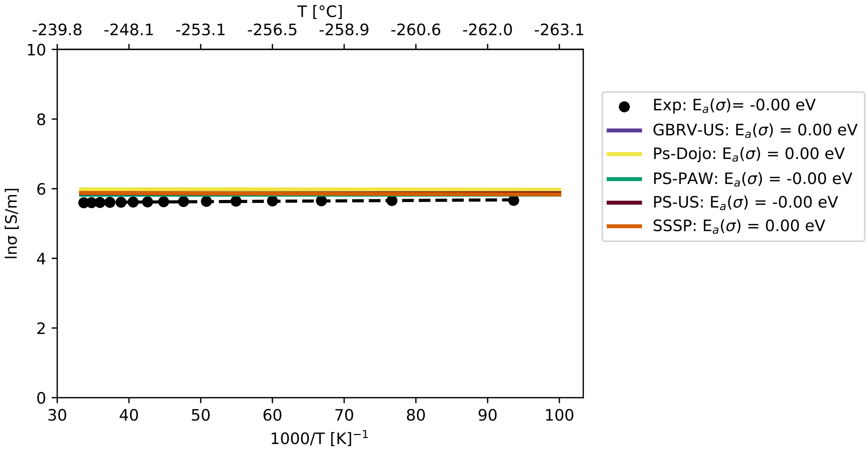

(c) PBE+U_{AFLOW}

Figure S45: The electrical conductivity (σ) of V_2O_3 vs temperature. The pseudopotential libraries are compared separately for each of the indicated DFAs. The activation energies are shown in the legend for the PPs libraries (solid lines) and experimental study (black points) [Carter et al. [22]].

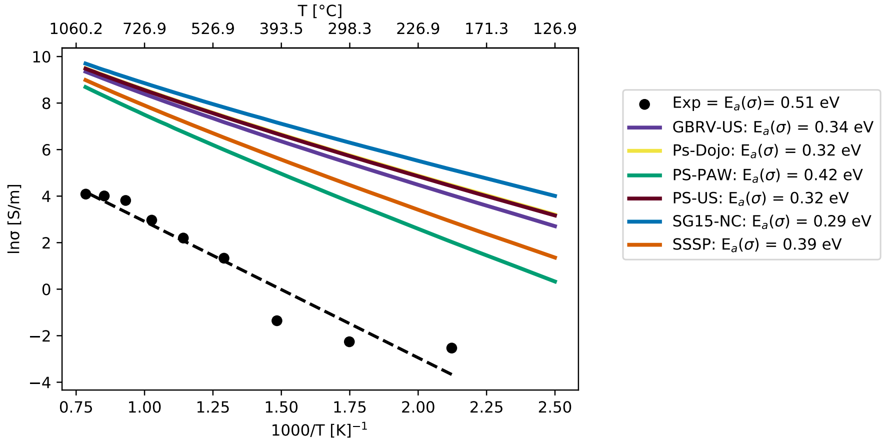

(a) PZ

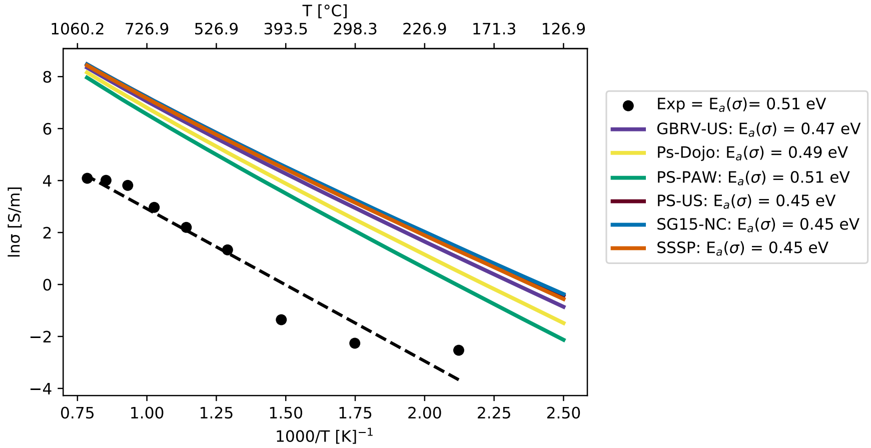

(b) PBE

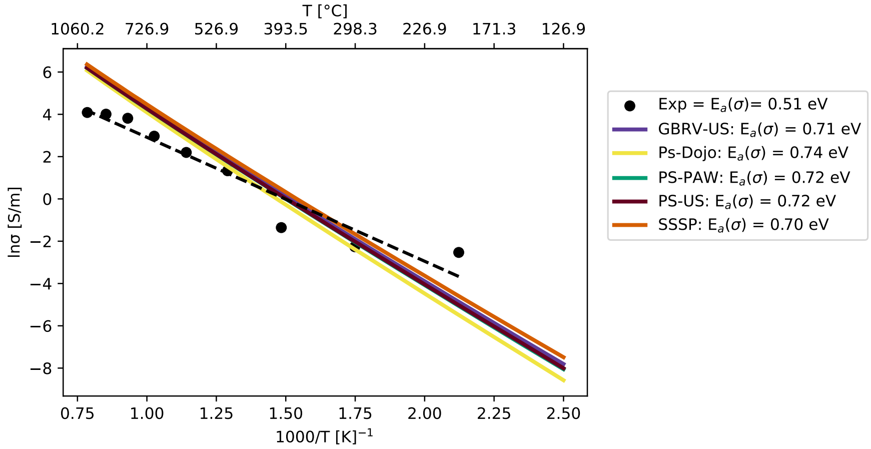

(c) PBE+U_{AFLOW}

Figure S46: The electrical conductivity (σ) of ZnO vs temperature. The pseudopotential libraries are compared separately for each of the indicated DFAs. The activation energies are shown in the legend for the PPs libraries (solid lines) and experimental study (black points) [Kim et al. [9]].

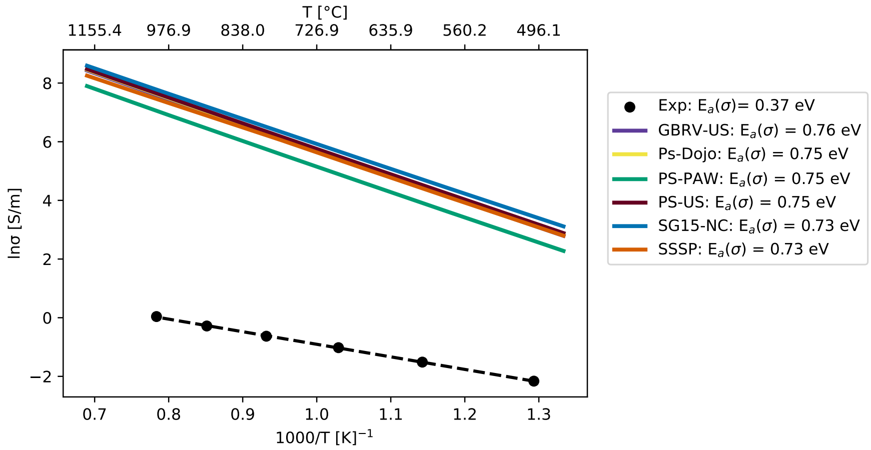

(a) PZ

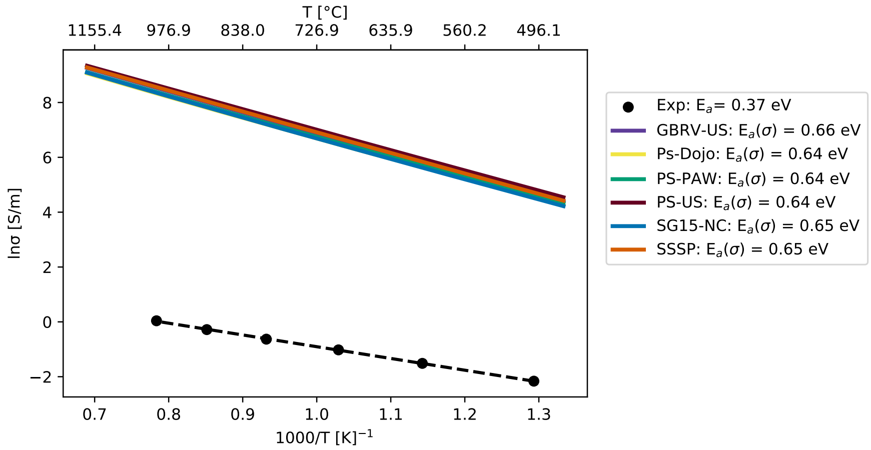

(b) PBE

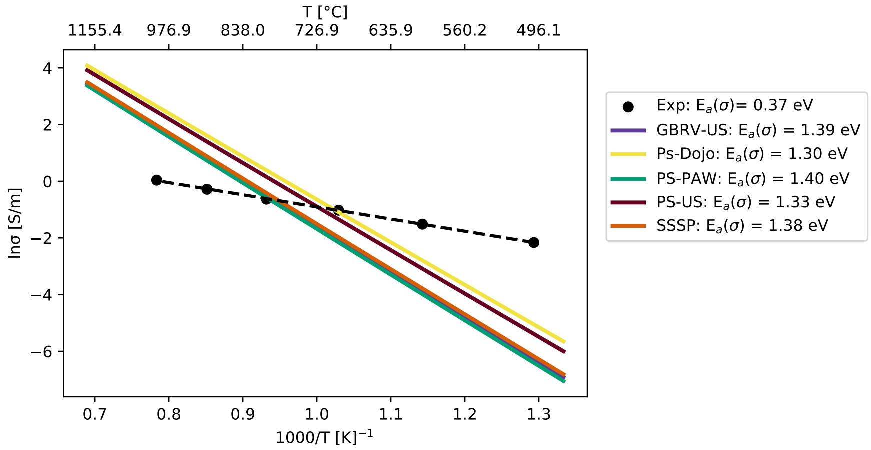

(c) PBE+U_{AFLOW}

Figure S47: The electrical conductivity (σ) of Cr_2O_3 vs temperature. The pseudopotential libraries are compared separately for each of the indicated DFAs. The activation energies are shown in the legend for the PPs libraries (solid lines) and experimental study (black points) [Nagai et al. [11]].

2.3.4 Computational cost: BoltzWann vs BoltzTrap

In the section below, we compare the computational performance of BoltzWann (Wannier-based transport) and BoltzTrap (Fourier-direct interpolation-based transport) using the same k-point grids for the same temperature range. BoltzWann has an additional cost associated with the Wannierization process, which BoltzTrap does not have. BoltzWann gives consistent results between the two k-point meshes (8x8x4 and 16x16x8), see Figure S48. This is not the case for BoltzTrap, especially for the lower temperatures.

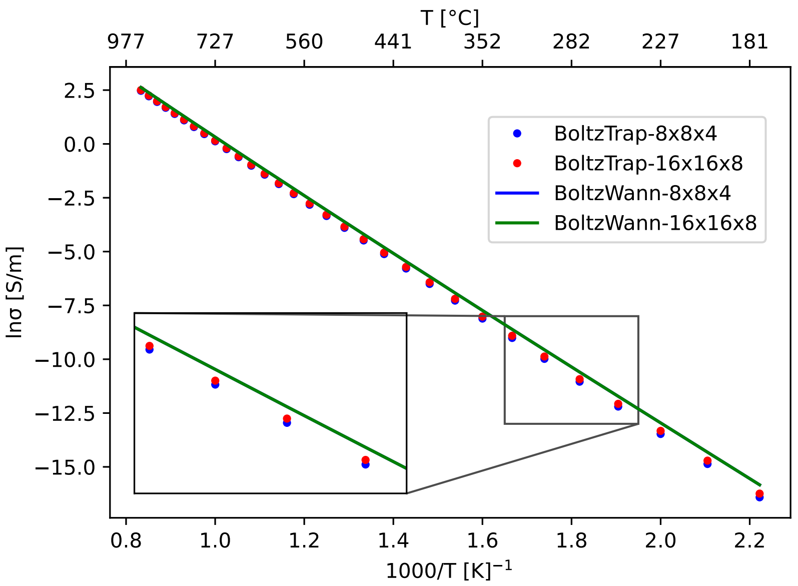

Figure S48: Validation of our work using BoltzWann code against BoltzTrap code for one representative system: TiO_2-A. Solid lines indicate results from BoltzWann, while dots represent results from BoltzTrap.

A comparison of the computational time between BoltzWann and BoltzTrap is shown in Table S7. The well-converged low k-point density BoltzWann calculation (8x8x4, blue line in Fig. S48) is faster than the high k-point density BoltzTrap calculation (16x16x8, red dots in Fig. S48), which would require even more k-points to be converged.

Table S7: Computational time comparison between BoltzWann and BoltzTrap workflows for the computation of the electrical conductivity of TiO_2-A.

	BoltzWann-8x8x4	BoltzWann-16x16x8	BoltzTrap-8x8x4	BoltzTrap-16x16x8
scf	21 s	73 s	21 s	73 s
nscf	241 s	1948 s	241 s	1948 s
w90-pp	0 s	0 s		
pw2wannier	47 s	339 s		
post-process	1191 s	3285 s	60 s	60 s
total	1500 s	5646 s	322 s	2082 s

References

(1) C. E. Calderon, J. J. Plata, C. Toher, C. Oses, O. Levy, M. Fornari, A. Natan, M. J. Mehl, G. Hart, M. Buongiorno Nardelli and S. Curtarolo, "The AFLOW standard for high-throughput materials science calculations", *Computational Materials Science*, 2015, **108**, 233–238, DOI: 10.1016/j.commatsci.2015.07.019.

(2) P. Umari and A. Pasquarello, "*Ab initio* Molecular Dynamics in a Finite Homogeneous Electric Field", *Physical Review Letters*, 2002, **89**, 157602, DOI: 10.1103/PhysRevLett.89.157602.

(3) R. Resta and D. Vanderbilt, in *Physics of Ferroelectrics*, Springer Berlin Heidelberg, Berlin, Heidelberg, 2007, vol. 105, pp. 31–68, DOI: 10.1007/978-3-540-34591-6_2.

(4) J. Otsuka, T. Ono, K. Inagaki and K. Hirose, "First-principles calculations of dielectric constants of C 20 bulk using Wannier functions", *Physica B: Condensed Matter*, 2006, **376-377**, 320–323, DOI: 10.1016/j.physb.2005.12.082.

(5) R. Warmbier, F. Mohammed and A. Quandt, "Optical and other material properties of SiO2 from *ab initio* studies", *Optical Engineering*, 2014, **53**, 071808, DOI: 10.1117/1.OE.53.7.071808.

(6) A. T. Onawole, M. S. Nasser, I. A. Hussein, M. J. Al-Marri and S. Aparicio, "Theoretical studies of methane adsorption on Silica-Kaolinite interface for shale reservoir application", *Applied Surface Science*, 2021, **546**, 149164, DOI: 10.1016/j.apsusc.2021.149164.

(7) I. Petousis, W. Chen, G. Hautier, T. Graf, T. D. Schladt, K. A. Persson and F. B. Prinz, "Benchmarking density functional perturbation theory to enable high-throughput screening of materials for dielectric constant and refractive index", *Physical Review B*, 2016, **93**, 115151, DOI: 10.1103/PhysRevB.93.115151.

(8) F. Ricci, W. Chen, U. Aydemir, G. J. Snyder, G.-M. Rignanese, A. Jain and G. Hautier, "An ab initio electronic transport database for inorganic materials", *Scientific Data*, 2017, **4**, 170085, DOI: 10.1038/sdata.2017.85.

(9) K. H. Kim, S. H. Shim, K. B. Shim, K. Niihara and J. Hojo, "Microstructural and Thermoelectric Characteristics of Zinc Oxide-Based Thermoelectric Materials Fabricated Using a Spark Plasma Sintering Process", *Journal of the American Ceramic Society*, 2005, **88**, 628–632, DOI: 10.1111/j.1551-2916.2005.00131.x.

(10) Y. Liu, Q. Chen, X. Du, X. Liu and P. Li, "Effects of substrate on the structure and properties of V2O5 thin films prepared by the sol-gel method", *AIP Advances*, 2019, **9**, 045028, DOI: 10.1063/1.5095718.

(11) H. Nagai, T. Fujikawa and K.-i. Shoji, "Electrical Conductivity of Cr2O3 Doped with La2O3, Y2O3 and NiO*", *Transactions of the Japan Institute of Metals*, 1983, **24**, 581–588, DOI: 10.2320/matertrans1960.24.581.

(12) S. K. Deb, "Physical properties of a transition metal oxide: optical and photoelectric properties of single crystal and thin film molybdenum trioxide", *Proceedings of the Royal Society of London. Series A. Mathematical and Physical Sciences*, 1997, **304**, 211–231, DOI: 10.1098/rspa.1968.0082.

(13) P. Knauth and H. L. Tuller, "Electrical and defect thermodynamic properties of nanocrystalline titanium dioxide", *Journal of Applied Physics*, 1999, **85**, 897–902, DOI: 10.1063/1.369208.

(14) T. Dittrich, J. Weidmann, F. Koch, I. Uhlendorf and I. Lauermann, "Temperature- and oxygen partial pressure-dependent electrical conductivity in nanoporous rutile and anatase", *Applied Physics Letters*, 1999, **75**, 3980–3982, DOI: 10.1063/1.125513.

(15) E. H. Greener, F. J. Barone and W. M. Hirthe, "Electrical Conductivity of Single and Polycrystalline Near-Stoichiometric Rutile in the Range 600° to 1400°C", *Journal of the American Ceramic Society*, 1965, **48**, 623–627, DOI: 10.1111/j.1151-2916.1965.tb14692.x.

(16) H. Tang, H. Berger, P. Schmid, F. Lévy and G. Burri, "Photoluminescence in TiO2 anatase single crystals", *Solid State Communications*, 1993, **87**, 847–850, DOI: 10.1016/0038-1098(93)90427-O.

(17) J. Pascual, J. Camassel and H. Mathieu, “Fine structure in the intrinsic absorption edge of TiO_2”, *Physical Review B*, 1978, **18**, 5606–5614, DOI: 10.1103/PhysRevB.18.5606.

(18) Z. Bodó and I. Hevesi, “Optical Absorption near the Absorption Edge in V_2O_5 Single Crystals”, *physica status solidi (b)*, 1967, **20**, DOI: 10.1002/pssb.19670200153.

(19) W. Y. Liang and A. D. Yoffe, “Transmission Spectra of ZnO Single Crystals”, *Physical Review Letters*, 1968, **20**, 59–62, DOI: 10.1103/PhysRevLett.20.59.

(20) M. M. Abdullah, F. M. Rajab and S. M. Al-Abbas, “Structural and optical characterization of Cr2O3 nanostructures: Evaluation of its dielectric properties”, *AIP Advances*, 2014, **4**, 027121, DOI: 10.1063/1.4867012.

(21) L. Ben-Dor and Y. Shimony, “Crystal structure, magnetic susceptibility and electrical conductivity of pure and NiO-doped MoO2 and WO2”, *Materials Research Bulletin*, 1974, **9**, 837–844, DOI: 10.1016/0025-5408(74)90120-2.

(22) S. A. Carter, T. F. Rosenbaum, P. Metcalf, J. M. Honig and J. Spalek, “Mass enhancement and magnetic order at the Mott-Hubbard transition”, *Physical Review B*, 1993, **48**, 16841–16844, DOI: 10.1103/PhysRevB.48.16841.
